# A distinct E dimer epitope underlies selective recognition by a protective human West Nile virus antibody

**DOI:** 10.1038/s44319-026-00851-z

**Published:** 2026-06-29

**Authors:** Baldeep Khare, Charles-Adrien Arnaud, Thomas Klose, James E Crowe Jr, Richard J Kuhn

**Affiliations:** 1 Department of Biological Sciences, Purdue University, West Lafayette, IN USA; 2 Vanderbilt Center for Antibody Therapeutics, Nashville, TN USA; 3 Department of Pediatrics, Vanderbilt University Medical Center, Nashville, TN USA; 4 Department of Pathology, Microbiology and Immunology, Vanderbilt University Medical Center, Nashville, TN USA; 5 Purdue Institute of Inflammation, Immunology and Infectious Disease, West Lafayette, IN USA

**Keywords:** Immunology, Microbiology, Virology & Host Pathogen Interaction, Structural Biology

## Abstract

Human outbreaks of West Nile virus (WNV) are an imminent threat in North America, with many annual infections and numerous cases of severe neuroinvasive disease. There are no licensed treatments for WNV disease. Previous research identified WNV-86 as an ultrapotent neutralizing human antibody that binds domain II of the major envelope (E) glycoprotein in mature virions. Here, we report the structure of mature WNV in complex with the Fab of WNV-86, at a resolution of 3.8 Å, solved using cryogenic electron microscopy. Structure-based epitope mapping identifies a new class of E dimer epitope (EDE) antibodies that we designate as EDE3 antibodies. A partial overlap of WNV-86 and pre-membrane protein (prM) binding regions at more than one site ensures selective binding of WNV-86 to mature virions. The structure reveals the quaternary epitope of the neutralizing Fab and supports a model in which engaging both protomers of the dimer likely interferes with fusion-triggering rearrangements. This study identifies critical residues for binding, neutralization, and immune escape and clarifies the promise of this molecule for future immunotherapeutic interventions.

## Synopsis

This study provides a comprehensive structural characterization of the ultrapotent human WNV-86 antibody and identifies the EDE-3 class of flavivirus antibodies. This has broad implications for flavivirus immuno-therapeutics involving different antibody isotypes across serocomplexes.

- West Nile virus WNV-86 is the prototype of the EDE-3 class of flavivirus antibodies.
- Insertion of the HCDR-3 loop at a vulnerable site ensures binding of WNV-86 to mature virions.
- Structural characterization of the epitope elucidates acquisition of glycosylation in DII domain as a route for immune escape.
- The EDE3 module may be present across antibody isotypes and flavivirus serocomplexes.

## Abstract (web summary)

This study provides a comprehensive structural characterization of the ultrapotent human WNV-86 antibody and identifies the EDE-3 class of flavivirus antibodies. This has broad implications for flavivirus immuno-therapeutics involving different antibody isotypes across serocomplexes.

## Graphical abstract

**Figure:**
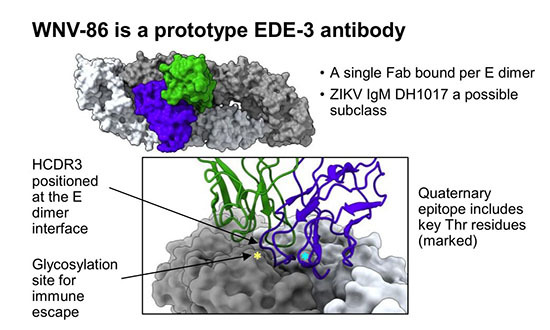

**Figure:**
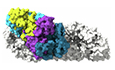

## Introduction

West Nile virus (WNV) is a phylogenetically diverse arbovirus that circulates in the Americas, Europe, Africa, the Middle East, Asia, and Oceania (Chancey et al, 2015; David and Abraham, 2016). WNV strains are classified into nine lineages with generally localized geographical prevalence (Bakonyi et al, 2006; Koch et al, 2024). Lineage 1 viruses comprise the largest group and predominate in North America, while lineage 2 strains are re-emerging in Europe (Bakonyi et al, 2006; Hernandez-Triana et al, 2014; Koch et al, 2024). Both lineages are causing increasing cases of severe neurological disease called West Nile neuroinvasive disease (WNND) in humans (Kleinschmidt-DeMasters and Beckham, 2015; Pacenti et al, 2020; Zehender et al, 2017). WNV infections are generally asymptomatic or result in mild fever-like malaise; however, WNND can manifest with meningitis, encephalitis, or paralysis and is associated with higher mortality in immunocompromised individuals (Debiasi, 2011; Pacenti et al, 2020; Racsa et al, 2014). While several vaccines are available to prevent WNV disease in horses, there are no licensed preventative measures or treatments for humans (Kaaijk and Luytjes, 2018; Kaiser and Barrett, 2019; Kuhn et al, 2023; Saiz, 2020). This situation is partly complicated by the sporadic nature of WNV outbreaks in humans, which limits the practicality of conducting clinical trials to test the efficacy of prophylactic or therapeutic candidates. However, structural investigations of neutralizing antibodies isolated from human survivors of flavivirus outbreaks can provide important insights into the nature of complex quaternary protective epitopes and reveal the molecular basis of antibody-mediated neutralization pertinent to the development of medical countermeasures for WNV disease.

The *Flaviviridae* family and the genus *Orthoflavivirus* consist of positive-stranded RNA viruses that include West Nile virus (WNV), Usutu virus (USUV), Japanese encephalitis virus (JEV), Zika virus (ZIKV), dengue virus (DENV), yellow fever virus (YFV), and tick-borne encephalitis virus (TBEV) (Postler et al, 2023). WNV, JEV, and USUV are members of the Japanese encephalitis (JE) serocomplex (Calisher et al, 1989; Kuno et al, 1998). Mosquitoes of the *Culex* genus are the primary vector for these viruses, and birds, especially members of the *Corvidae* family, are the reservoir. WNV can infect a broad range of animals, but horses and humans are dead-end hosts (Khare and Kuhn, 2022). The primary mode of transmission in humans is the bite of an infected mosquito, while less common routes of transmission include infections via organ transplantation, blood transfusions, breastfeeding, or vertical transmission (Khare and Kuhn, 2022). Transmission dynamics of WNV are complex and involve various virus, host, and environmental factors. However, since the introduction of lineage 1 or 2 strains in North America or Europe, respectively, the virus has steadily expanded geographically and evolved into more virulent strains, causing increasing cases of WNND (Kleinschmidt-DeMasters and Beckham, 2015). Similarly, a lineage 1b strain, designated WNV Kunjin, is primarily found in Oceania and is known to be an attenuated strain that does not cause disease in horses or humans (Adams et al, 1995; Hall et al, 2001). However, the emergence of the variant WNV NSW2011 strain in Australia, which causes severe disease and mortality in horses, exemplifies the ability of WNV strains of any lineage to evolve, expand, and cause sudden outbreaks in geographically distinct locations (Frost et al, 2012; Kleinschmidt-DeMasters and Beckham, 2015).

The flavivirus glycoprotein shell undergoes crucial acid-induced conformational changes during two events in the virus life cycle: virus maturation in the trans-Golgi network of an infected cell and endocytosis-mediated changes in the mature, infectious virus after virus entry into host cells (Mukhopadhyay et al, 2005). The E and pre-membrane (prM) proteins form the outer shell of the particle, with their transmembrane domains embedded in a host-derived membrane (Khare et al, 2021; Kuhn et al, 2002; Renner et al, 2021). The immature virion contains 60 copies of prM:E trimers protruding away from the virus surface, giving them a spiky appearance (Zhang et al, 2003). As the virus traverses the low-pH environment of the trans-Golgi, a conformational change exposes the furin cleavage site prior to prM cleavage into pr and M proteins by the host furin protease (Yu et al, 2008; Zhang et al, 2004). The trimers of E:prM heterodimers are thus reorganized into 90 E:pr/M heterodimers arranged in a head-to-tail fashion, which lie flat on the virus surface (Yu et al, 2008). Cleavage of prM renders the maturation process irreversible. Pr remains associated with E protein dimers at low pH and dissociates from the complex at neutral pH for most flaviviruses, but not in YFV (Crampon et al, 2023; Yu et al, 2008). Changes in electrostatic interactions and conformation of the glycan loop at neutral pH, from an “open” to a “closed” state, expel pr protein from the virus at neutral pH (Vaney et al, 2022).

Structural studies of flaviviruses, using x-ray crystallography or cryogenic electron microscopy, in their different conformational states, as well as of complexes of viruses bound with antibodies or Fabs, have advanced our understanding of virus maturation, pathogenesis, neutralization, and immune escape in the host (Mukhopadhyay et al, 2005). These studies have been instrumental in defining epitopes, especially the discontinuous or quaternary epitopes of neutralizing antibodies targeting E protein. Furthermore, such studies can define the molecular basis of neutralization of specific classes of antibodies, such as the E dimer epitope (EDE) class of antibodies (Barba-Spaeth et al, 2016; Dejnirattisai et al, 2015). Potently neutralizing antibodies isolated from B cells of human convalescent patients are especially informative in defining determinants of virus neutralization and strategies of immune escape. Previously, we functionally characterized the WNV-86 antibody, isolated from a survivor of the Dallas (USA) outbreak of lineage I WNV in the summer of 2012 (Goo et al, 2019) and defined the domains containing the epitope, escape mechanisms, and some critical residues involved in binding. Here, we structurally characterize the WNV-86 antibody in complex with WNV, map the antibody epitope, and elucidate the molecular basis of previously reported observations. We define a new class of highly potent E dimer antibodies, which we designate EDE3 antibodies, and report their presence across antibody isotypes.

## Results

### WNV-86 epitope

**Figure 1:**
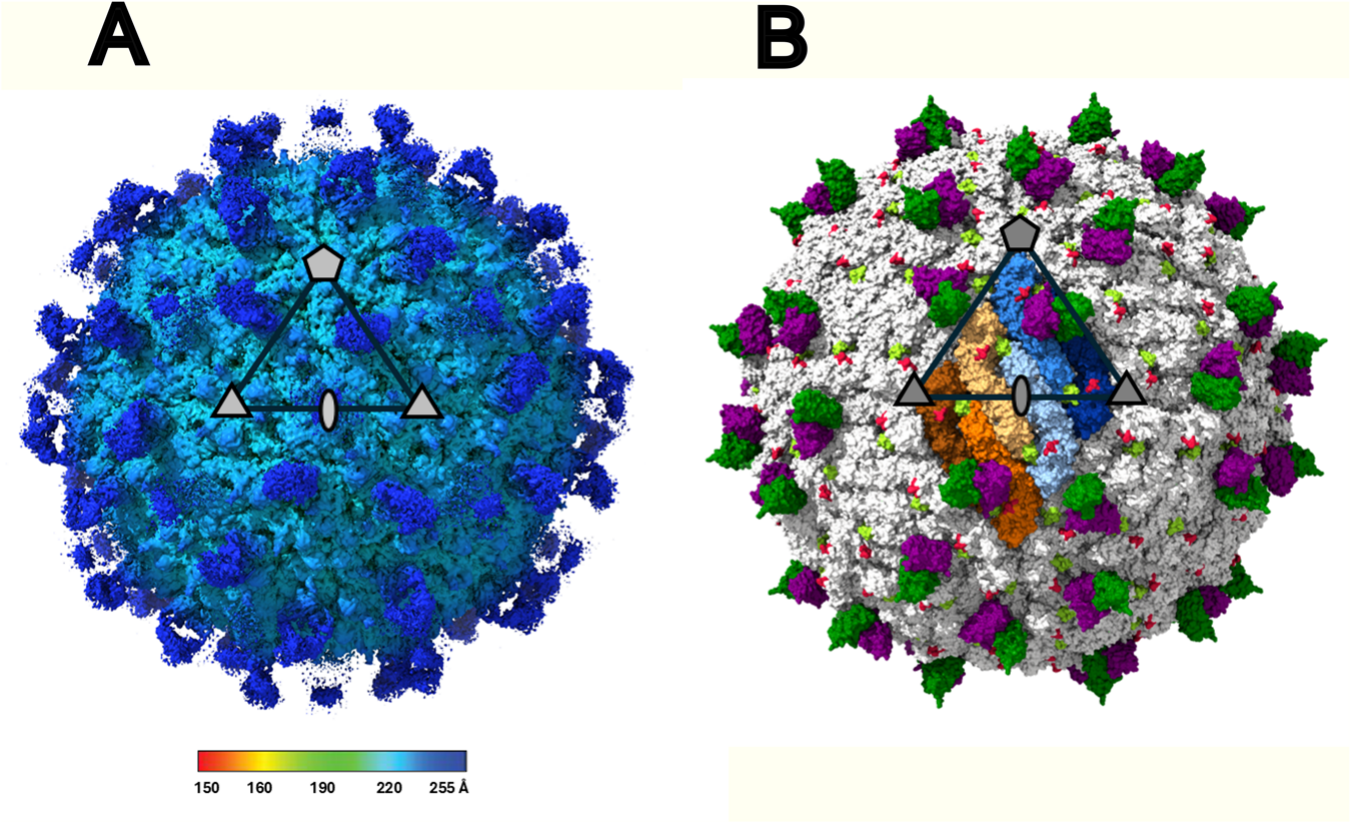
Cryo-EM reconstruction and model of the mature WNV virion with bound WNV-86 Fab. (**A**) The cryo-EM reconstruction of the full virion, viewed down the icosahedral twofold axis. The asymmetric subunit (ASU) is marked, and the map is color-coded according to radial distance (Å) as shown. The WNV-86 Fab density is observed for two of the three sites in the ASU. (**B**) A view of the model of the virion with the WNV-86 Fab bound at the general dimer position, viewed in the same icosahedral orientation. The Fv domains of the heavy and light chains of the Fab are colored purple and green, respectively. Three E proteins, from two adjacent ASUs, are shown in shades of blue (chains A, C, and E, colored light, medium and dark blue, respectively) or orange (A′, C′, and E′, depicted in light, medium and dark orange, respectively). The fusion loop (amino acid residues 98–110) is highlighted in lime green. Amino acid residues 154–156, which form the glycosylation motif in the glycan loop of E, are highlighted in crimson. The fusion and glycan loops do not colocalize with the Fab interface. Source data are available online for this figure.

**Figure 2:**
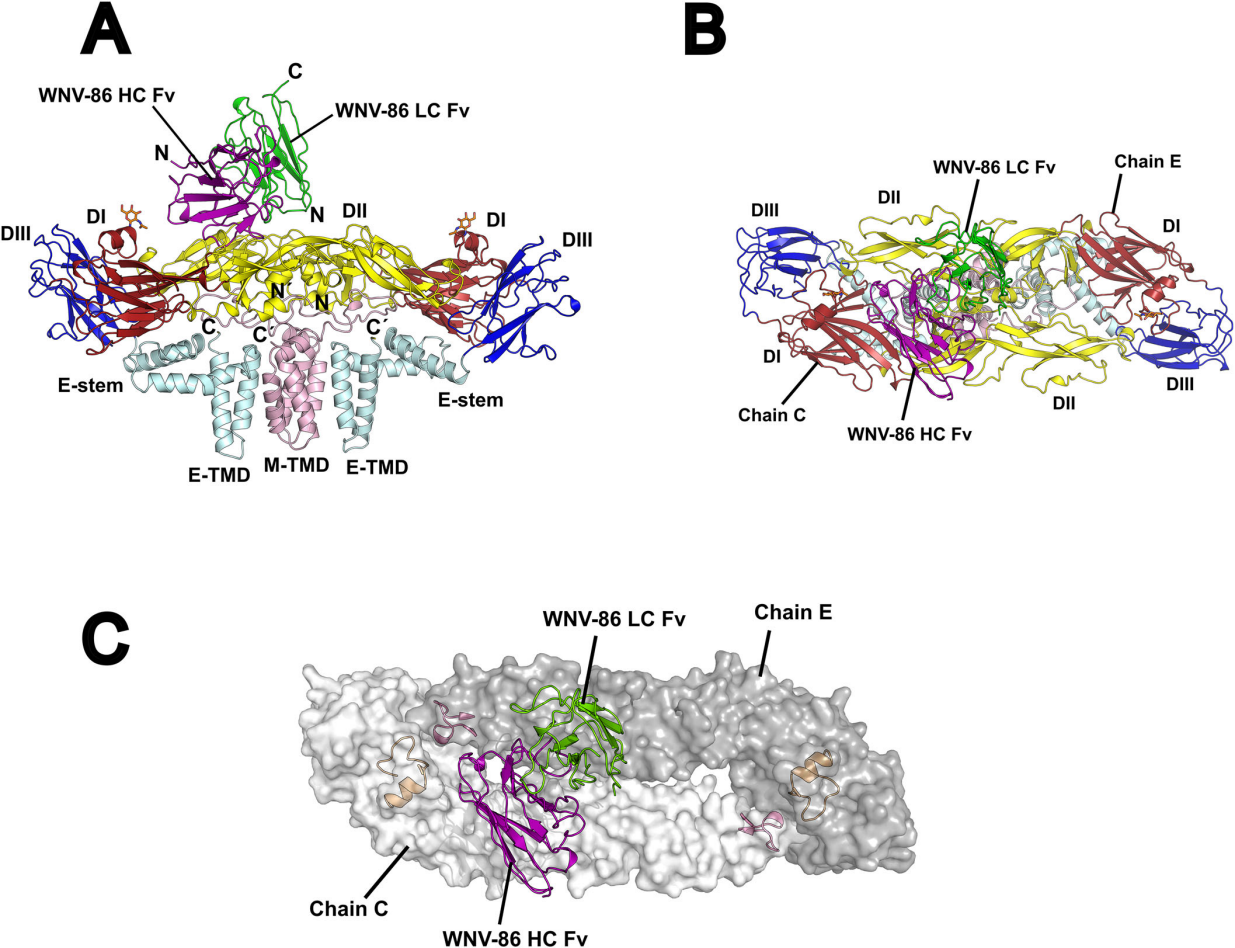
Model of the WNV E:M tetramer with the bound WNV-86 Fab. (**A**) A ribbon model of the E:M tetramer as viewed in a cross-sectional plane of the virion. (**B**) The same model as viewed perpendicular to the surface of the virus particle. (**C**) Surface representation of the E dimer with the fusion loop and the glycan loop of E proteins and the Fab Fv domains in cartoon representation. The two chains of the E dimer are shown in gray and light-gray. The Fv domains of the heavy and light chains of WNV-86 Fab are purple and green, respectively. The Fv domains lie away from the fusion and glycan loops. Source data are available online for this figure.

An icosahedral 3D reconstruction of the complex of WNV and Fab of WNV-86 antibody was determined at a resolution of 3.8 Å, using a Fourier shell correlation (FSC) estimation at 0.143 (Fig. 1A,B; Appendix Fig. S1A–F and Appendix Table S1) (Scheres and Chen, 2012). The binding architecture of the WNV-86 Fab on the virion reveals density for the Fab at the E dimers; no density corresponding to the Fab was observed at the icosahedral fivefold or threefold axes (Fig. 1A). The mature flavivirus particle displays a pseudo *T* = 3 symmetry with three E proteins in the asymmetric subunit (ASU) and hence three potential binding sites for WNV-86 antibody (Fig. 1B) (Khare et al, 2021; Kuhn et al, 2002) Chains C and E of the E proteins form the general dimer and the icosahedral dimer is formed by chain A of one asymmetric subunit (ASU) with chain A′ of the adjacent ASU (Fig. 1B) (Zhang et al, 2004). The occupancy of the WNV-86 Fab was low; however, sufficient density of the Fv domains at the general dimer position (with an occupancy around 30%, Appendix Fig. S2A,B) enabled fitting a model at this site. The density at the general dimer position showed that the antibody displayed preferential binding, and a second Fab binding in the opposite direction would be sterically obstructed. Two of the three sites in the ASU support WNV-86 binding; hence, ~1.5 binding sites per ASU would accommodate at most 90 sites on the mature WNV particle. Localized reconstruction of the ASU failed to improve resolution; however, it displayed better continuity for the backbone Cα chain density (Appendix Fig. S1E,F). In this study we focus on the general dimer for unambiguous characterization of the WNV-86 epitope at this position. The interface of WNV-86 Fab with the virus spanned both protomers of the E dimer, with the variable domains of heavy or light chains of the Fab (HC or LC Fv domains, respectively) positioned above E chains C or E, respectively (Fig. 2A,B). The Fab domains were positioned across the E dimer at an angle, between the DI/DIII domains on one side and the twofold axis on the other. The buried surface areas for HC and LC Fv domains with the corresponding chains C or E are 421 or 265 Å^2^, respectively. The area of the WNV-86 HC Fv domain interface with chain E is greater, at 494 Å^2^.

**Figure 3:**
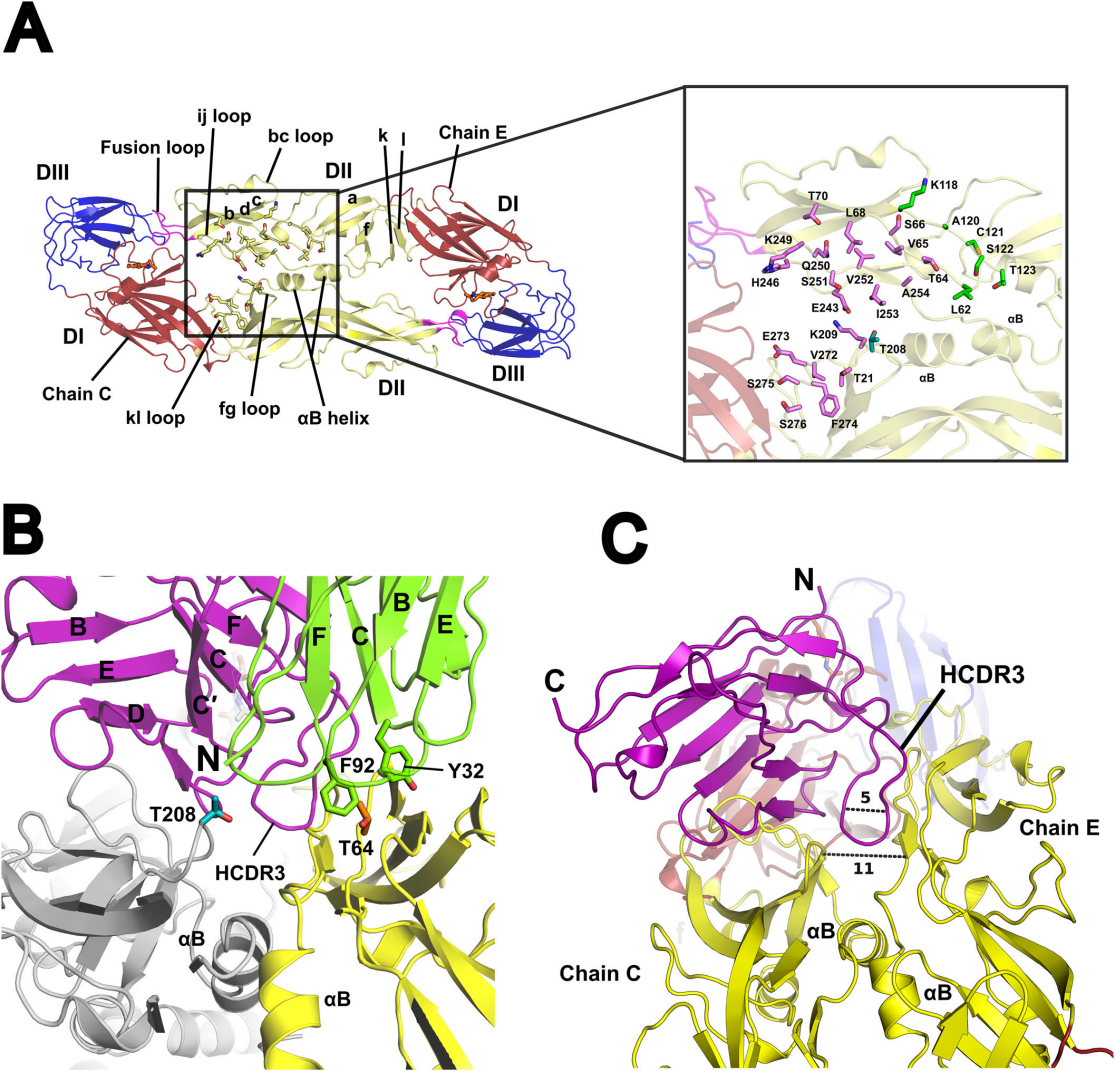
The WNV-86 epitope and the HCDR3 loop. (**A**) E protein residues that lie within 4.5 Å from WNV-86 Fv domains are shown in sticks, and the secondary structures or loops that they localize on are labeled. The inset shows the same residues labeled and color-coded for the chain of the Fv domain that are within 4.5 Å: E residues are colored violet for Fab heavy chain (Fab HC Fv) and green for the Fab light chain. (**B**) The interface of WNV-86 Fab and E protein chains E (yellow) and C (gray) shows the HCDR3 loop positioned in the groove, close to chain E. Thr64 (teal) of chain C and Thr208 (orange) of chain E lie roughly across the groove, with the HCDR3 loop in between. The heavy and light chain Fv domains are colored as described previously. (**C**) The width of the E dimer groove (Å) is roughly twice that of the HCDR3 loop, and the model shows that no major conformational changes result from the Fab binding. Source data are available online for this figure.

**Figure EV1:**
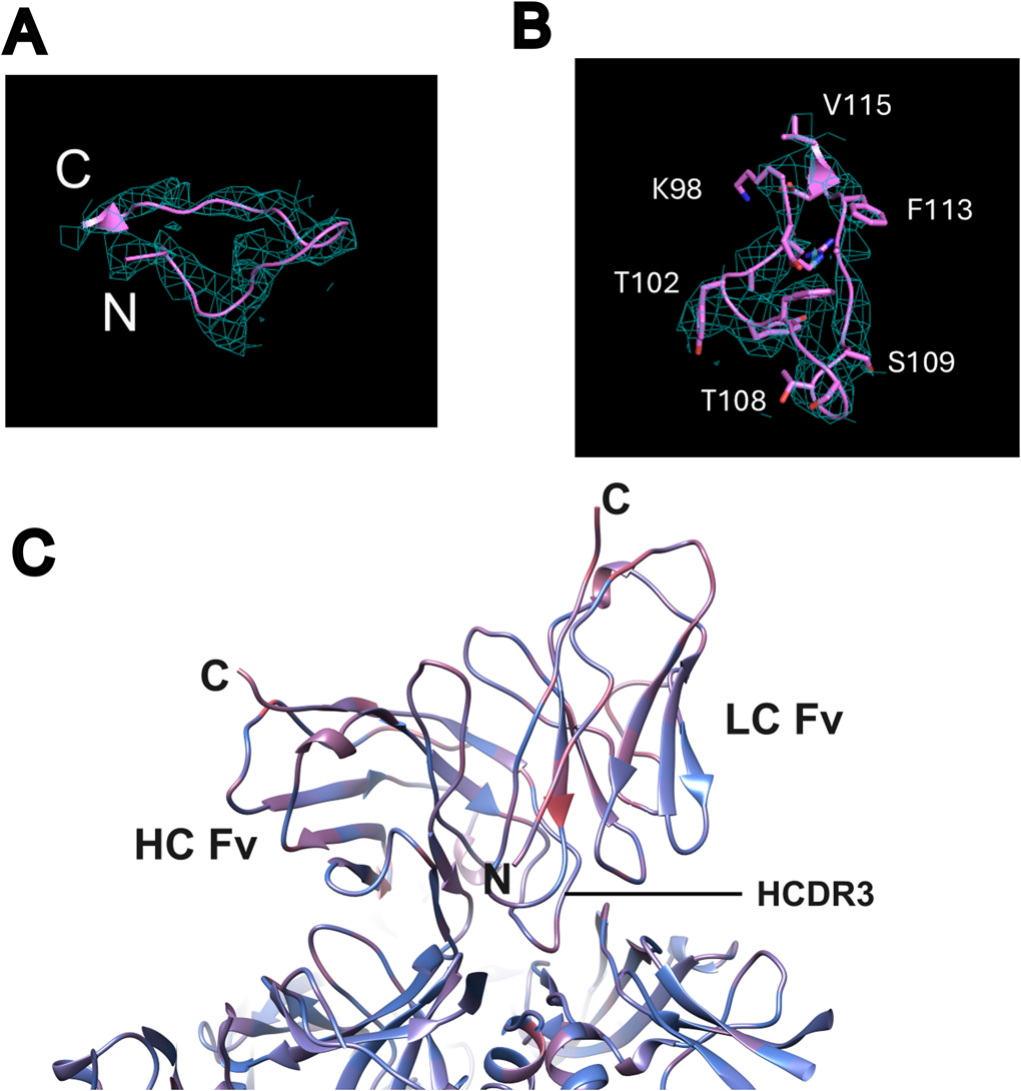
Density for the HCDR3 loop and model-to-map quality. (**A**) The density around the HCDR3 loop is shown with the loop represented in cartoon (sigma 1.0). (**B**) Side chains of some of the residues within the loop are depicted in sticks and labeled. The side chain density for bulky side chains corroborates a reasonable fit of both residues and the loop in the cryoEM map. (**C**) The Q score indicates the resolvability of the atoms in the map. An overall score of 0.52 and the model indicate that the residues and side chains are well resolved. Source data are available online for this figure.

**Figure EV2:**
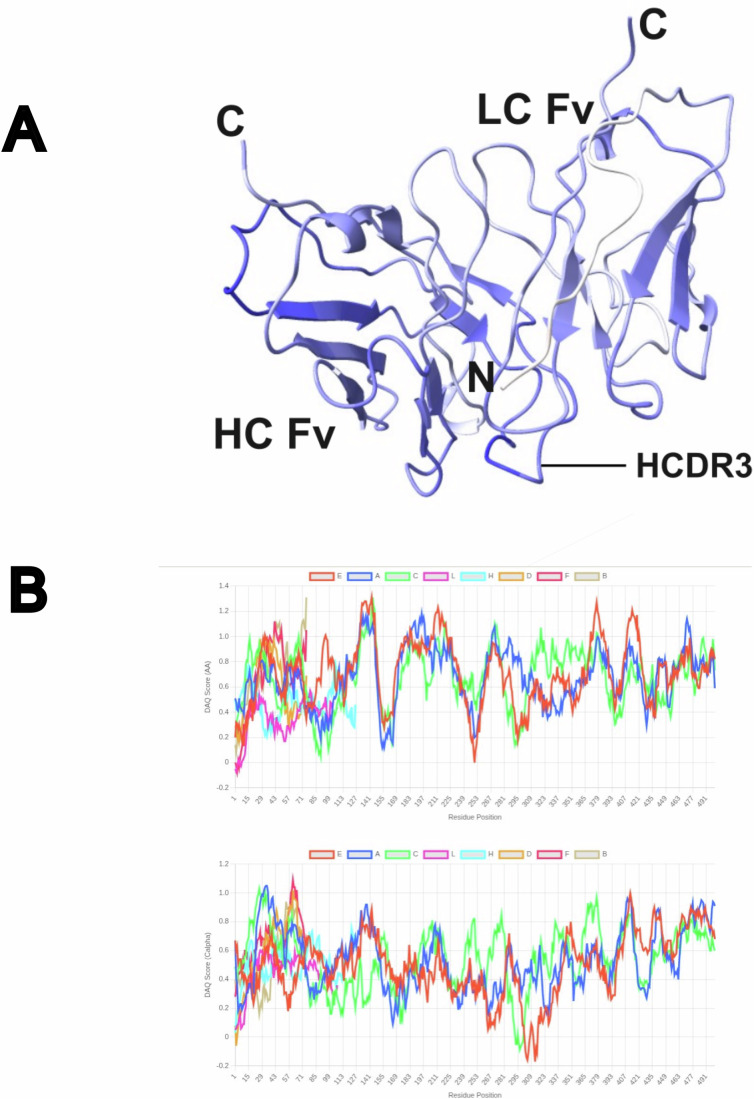
The DAQ score of WNV-86 Fab. (**A**) The DAQ score quantifies the local quality of the model and can identify wrongly modeled residues and regions. The score is from −1 (red), 0 (white) to +1 (blue). The DAQ score on the model of the E and Fab complex shows a reasonably accurate fit and quality for both E and the Fab, and especially for the HCDR3 loop region. (**B**) The graph shows the DAQ score by residues in different chains in the complex. The positive values for both Fv domains indicate a reasonably accurate fit of the Fab model in the cryoEM map. Source data are available online for this figure.

The interface of the Fab with E showed a discontinuous epitope that localized to the DII domain (Fig. 3A). Of the three complementarity-determining regions (CDRs) of the heavy chain of WNV-86, referred to hereafter as HCDR1, 2 or 3 (residues 26–35, 50–66 or 99–115, respectively), residues on HCDR2 and HCDR3 are positioned for several interactions with the two chains of the dimer. Local resolution at the Fab-glycoprotein interface varies between 3.6 and 4.5 Å (Appendix Fig. S3A–C); however, better loop density from the localized reconstruction of the ASU enabled modeling of the loop and assessment of potential interactions at this interface (Fig. 3B, C and EV1A,B). Resolvability of atoms in the map and presence of any incorrectly placed atoms in the model were assessed by analyzing the Q score (Fig. EV1C) or the DAQ score of the model (Fig. EV2A,B), which confirmed the accurate fit of the loop and the model in the density (Fig. EV1A,B) (Pintilie et al, 2025; Terashi et al, 2022). Aromatic and hydrophobic residues Tyr102 and Trp53 from HCDR loops and Tyr32, Ile30, and Phe92 from LCDR loops (LCDR1, 2, or 3, residues 24–34, 50–56 or 89–97, respectively) surrounded E residue Thr208 or Thr64 of chains C or E, respectively (Fig. 3A–C). For E protein chain C, the antibody CDR loops were positioned favorably for interactions with WNV E protein strands f, g, and k, and the loops connecting these strands: the Thr208 residue of E was positioned between His57 of HCDR2 on one side and Phe104 of HCDR3 on the other. Thr208 is also localized within 4 Å of Ile58 and Ser106 from the two HCDR loops. Other E residues that localized close to the Fab were Phe274 and Ser276. Phe274 is conserved in the JE virus serocomplex, except for JEV (Appendix Fig. S4A–C). LCDR3 faced E residues Val252 and Val65. Many of the E residues mentioned above are conserved across the JE virus serocomplex (Appendix Fig. S4A,B). All three loops of the light chain variable domain of the Fab were positioned above different regions of E chain E (Figs. 2 and 3A). LCDR1 and 3 faced strand b; LCDR2 and 3 faced E strands a-d, and E residues Leu62, Thr64, Ser66, and Ser122. However, L chain residues were at a greater distance for direct interactions (Fig. 3). Thus, the primary interactions of E were with the Fv domains of the heavy chain and involved both protomers of the E dimer, with more interactions predicted with chain E of the dimer. This supports the observation by Goo et al that WNV-86 Fab can bind to an E monomer in solution (Goo et al, 2019).

### The WNV-86 epitope compared with the epitopes of known intra-dimer antibodies

**Figure 4:**
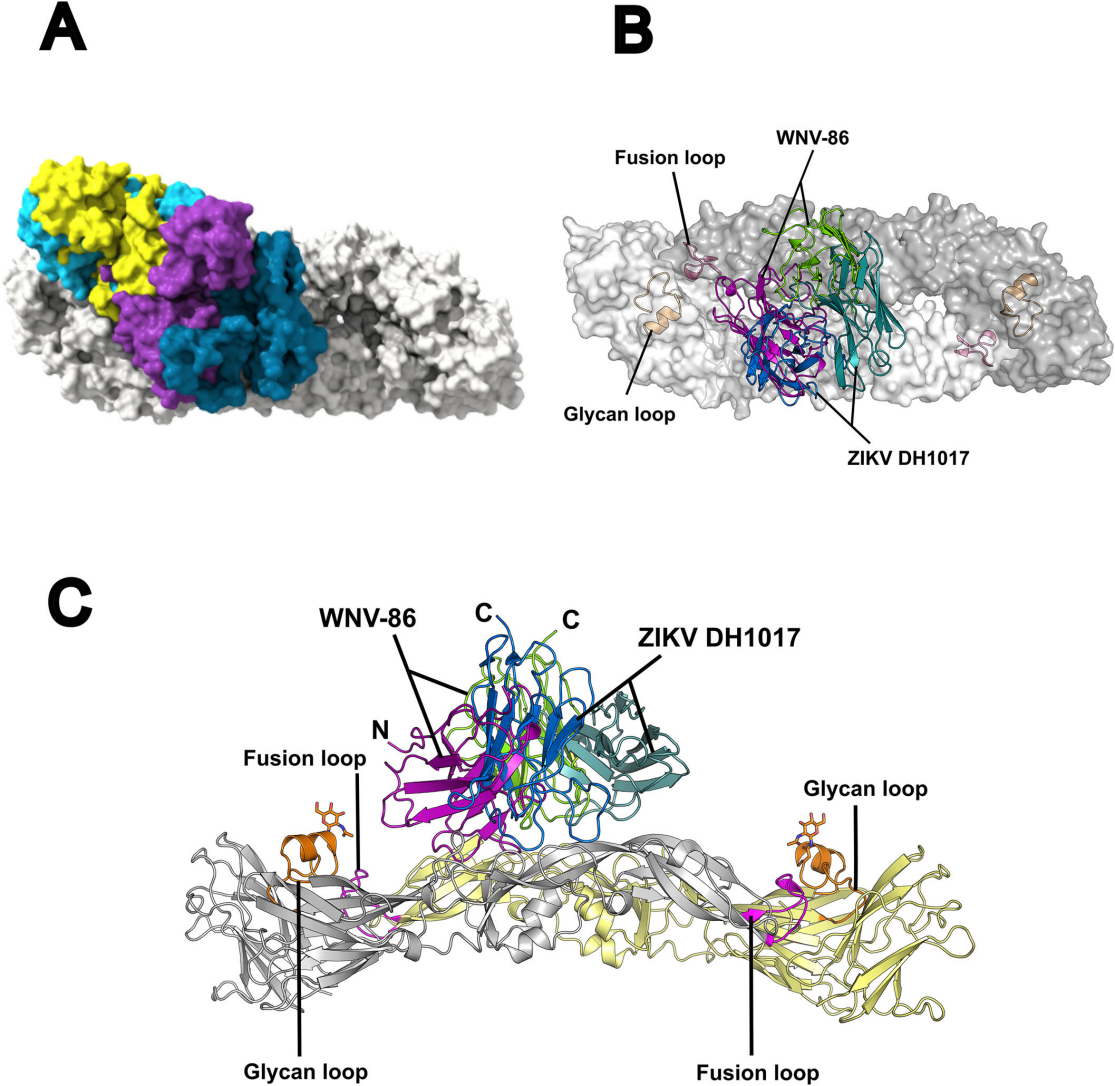
Interaction interface of the E-dimer epitope (EDE) antibodies. (**A**) Surface representation of the E dimer (WNV) with bound WNV-86 Fab (purple) and superposition of C8 EDE1 Fab (cyan), A11 EDE2 Fab (yellow), and ZIKV DH1017 (teal) shows the distinct binding regions. (**B**) Surface representation of the WNV E dimer with bound WNV-86 (ribbons, colored as described previously) and superposed ZIKV DH1017 Fab (blue and teal) shows the Fv domains of both EDE3 antibodies localized close to the E dimer twofold axis, and away from the fusion and glycan loops. (**C**) A side view of WNV E dimer ectodomain (chains C and E in gray or yellow, respectively) with bound WNV-86 (shown in ribbons and colored as described previously) and superposed ZIKV DH1017 (chains in blue and teal) shows the variations in CDR loops and the interaction interface between the two EDE3 antibodies. The fusion or glycan loops are highlighted in magenta or orange, respectively. Source data are available online for this figure.

The WNV-86 Fab interface, and hence the epitope, was limited to DII; interface residues within 4.5 Å were localized on both protomers of the dimer (Figs. 2A and 4A). This contrasts with the epitopes of E dimer epitope class 1 or 2 (EDE1 or EDE2) antibodies, for which the epitopes localize to a large area of DII from a single protomer and include the fusion loop, and the DI and DIII domains of the opposite protomer. The antibodies, therefore, occupied the ‘nook’ formed between DI and DIII or chain C and DII of chain E and localized over the fusion loop (Fig. 4A). Residues of DII targeted by the two known EDE classes of antibodies, represented by DENV mAb C8 (PDB ID 4UTA) and DENV mAb A11 (PDB ID 4UTB) were the same, and the epitopes differed in the residues involved in the DI and DIII domains (Gouet et al, 2003; Notredame et al, 2000). In contrast, for antibody WNV-86, residues from DI and DIII did not contribute to the epitope. EDE1 and EDE2 antibodies also differed in their requirement for the 150 loop; while the loop is dispensable for EDE1 antibodies, it is essential for the binding of EDE2 antibodies (Rouvinski et al, 2015). The role of mutations of specific residues on this loop for the binding of antibodies has been described (Goo et al, 2019). We observed density for the first NAG at N154, and the 150 loop was ordered with continuous density for both chains C and E of the E protein. The conformation of the 150 loop remains unchanged between the model of the complex (present study) and the WNV reconstruction determined at a resolution of 2.9 Å reported previously (PDB ID: 7KVA) (Hardy et al, 2021), suggesting that the WNV-86 Fab neither interacted with nor affected the conformation of the 150 loop.

The structure of ZIKV in complex with the Fab of ZIKV DH1017 showed a binding site architecture that is similar to that observed for WNV-86 binding (Fig. 4B,C) (Singh et al, 2022). ZIKV DH1017 is a potently neutralizing IgM isotype human antibody isolated from the B cells of a pregnant woman infected with ZIKV and shown to neutralize ZIKV but not DENV (Singh et al, 2022). A ZIKV DH1017 Fab-bound virus structure revealed binding to DII of both protomers on the E dimer (Fig. 4B,C). The ZIKV DH1017 Fab binding differed positionally from WNV-86 Fab; however, both Fabs revealed a predominant variable domain interface with one of the two protomers and formed a greater contact area with only one protomer (Fig. 4B,C). The CDR loops of ZIKV DH1017 formed a distinct contact interface from that observed for WNV-86; they did not occupy the E dimer groove, and two CDR loops were pointed toward the ZIKV E surface (Fig. 4B,C).

### Overlap with prM

**Figure 5:**
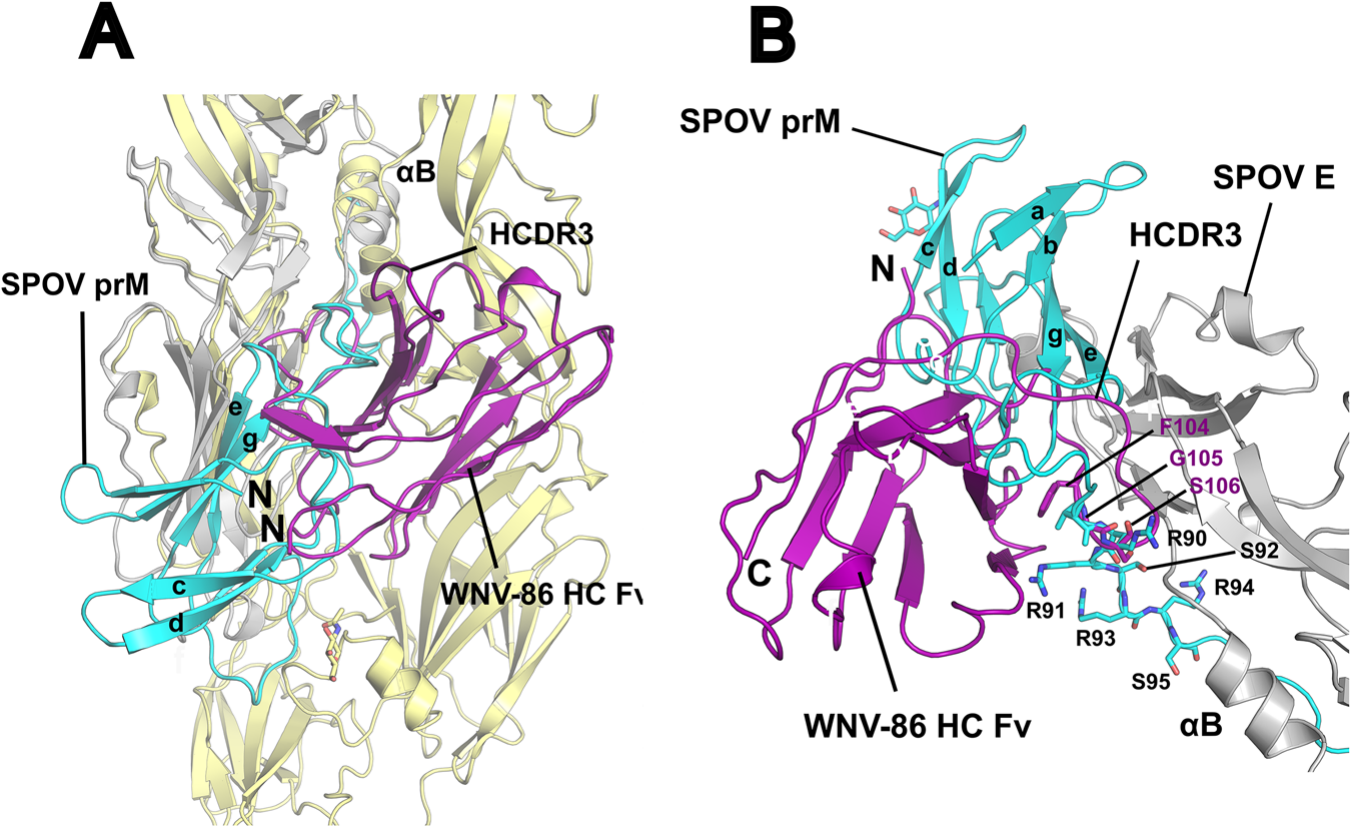
Overlapping binding sites of prM and WNV-86. (**A**) A view of the model of the WNV E dimer (gray) showing the overlap of bound WNV-86 Fab (purple: heavy chain variable domain) and the superposed Spondwenii virus (SPOV) immature E with bound prM (cyan, PDB ID: 6ZQI) shows clashes of prM loops with the heavy chain Fv domain CDR loops. (**B**) The same model shown only with the SPOV E (gray) is viewed at the furin cleavage motif of the prM and shows the overlap of two residues from prM and the WNV-86 heavy chain Fv domain (purple; residues represented in sticks and labeled in purple), with the superposed SPOV prM in cyan. The furin cleavage motif on prM is depicted in sticks, and these residues are labeled in black. Source data are available online for this figure.

**Figure EV3:**
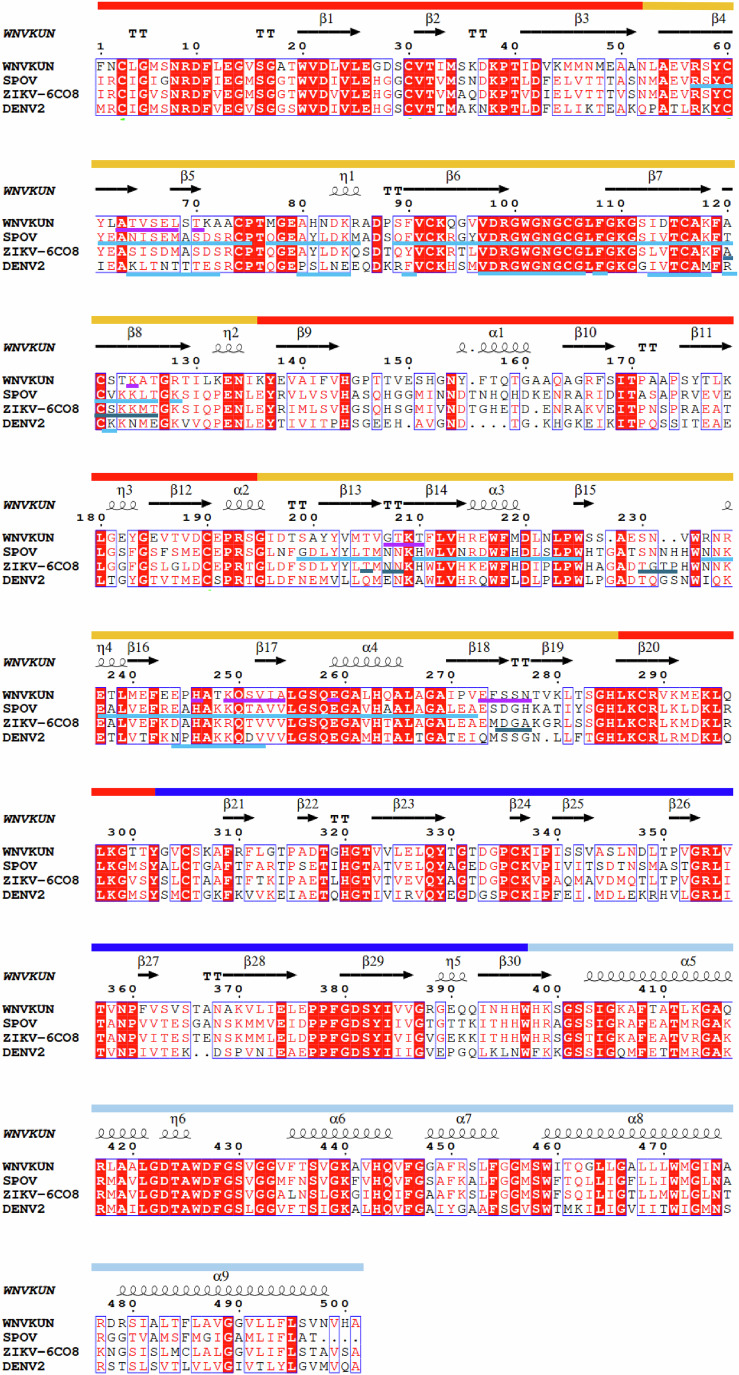
Interface residues mapped on E sequences. Sequences of E proteins from WNV, SPOV, ZIKV, and DENV2 are aligned using ESPript 3.0. Interface residues (within 4.5 Å contact distance) of WNV with WNV-86 are underlined in purple. The E residues for recombinant pr (DENV2, PDB code: 3C6E) and prM (SPOV, PDB code: 6ZQI) are underlined in cyan. The contact residues in ZIKV E for ZIKV DH1017 are underlined in teal. A colored bar above the sequences represents domains I-III (red, yellow, or blue, respectively) for WNV E protein. Source data are available online for this figure.

The HCDR3 loop was lodged between the E dimer, which was also the site for prM association with the E protein. Previous research identified preferential binding of WNV-86 to mature virions and predicted the possibility of overlap of the binding sites of WNV-86 and prM. Therefore, we investigated the secondary structures and interface residues contact of WNV-86 (this study) and prM binding in this region (Goo et al, 2019). Several structures, either of recombinant E protein complexed with pr protein or structures of the immature virus-containing prM, revealed varying details of the binding interface of pr/prM associated with E protein. Collectively, these structures revealed the position of the furin cleavage motif (Anastasina et al, 2024; Li et al, 2008; Newton et al, 2021; Renner et al, 2021). The sequence identity of prM proteins of WNV with those of DENV2, ZIKV, or Spondweni virus (SPOV) is approximately. 36, 45, or 50 percent, respectively. However, for SPOV, this conservation localizes largely to the transmembrane region and sporadically elsewhere in the protein (Appendix Fig. S4) (Renner et al, 2021). The cryo-EM structure of SPOV (PDB ID: 6ZQI) and the crystal structure of DENV2 E protein with pr bound (PDB ID: 3C6E) were determined at resolutions of 3.8 Å or 2.2 Å, respectively. These models were used as a reference for identifying possible determinants for the placement of the WNV-86 HCDR loop. The structure of prM consists of a seven-stranded β domain, followed by a nine-residue-long segment (residues 81-89 and containing His83 and Lys84, hereafter called the HK-loop; PDB ID: 6ZQI) that forms a loop preceding the furin cleavage motif (RR^91^SRR^94^S, SPOV numbering). This is followed by a long segment that forms the N-terminus of the M protein after furin cleavage, and then the transmembrane domain. The presence of prM will occlude the binding of WNV-86 Fab. Superimposition of SPOV E protein (PDB ID: 6ZQI) with the E protein in the complex structure of WNV with WNV-86 Fab showed that three loops from prM, occluded WNV-86 binding: residues on the HK-loop clashed with those on the LCDR3 loop and HC Fv domain β strands 4 and 5; prM β 4/5 and β 5/6 loops sterically hindered HCDR1 and HCDR3 (Fig. 5A). The placement of HCDR3 in the dimer groove forms the third site of occlusion; prM residues Thr^89^-Arg^90^ roughly overlapped with HCDR3 residues Gly^105^-Ser^106^ (Figs. 5B and EV3); hence, HCDR3 localized close to the same site as the prM furin cleavage segment. Arg90 is part of the furin cleavage motif, R^90^RSRRS^95^ (Renner et al, 2021). WNV-86 Ser106 and SPOV-prM Arg90, which overlapped in position in the superposed models, may represent a critical anchoring point in the E dimer.

### Residues essential for immune escape

**Figure 6:**
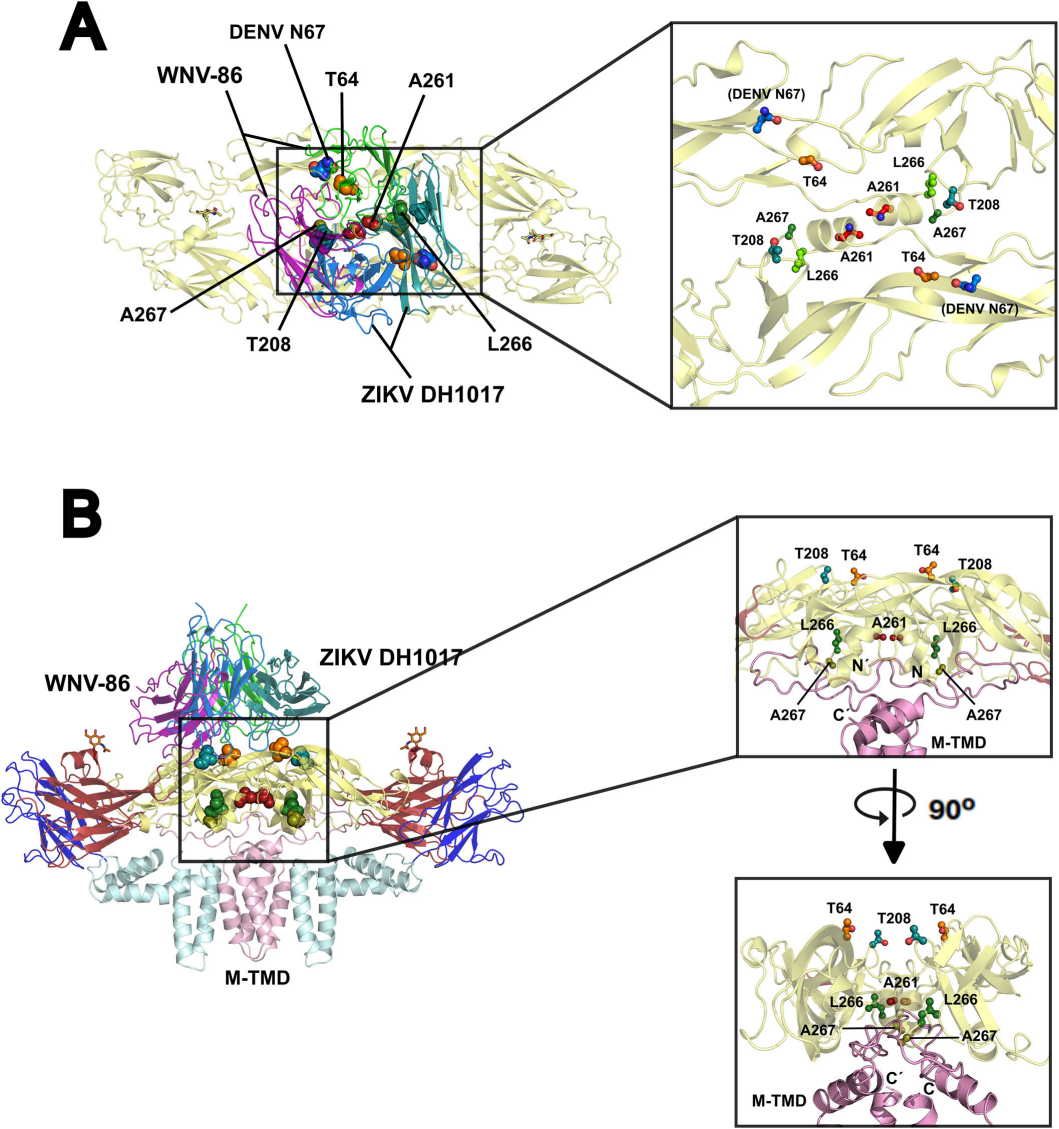
Residues essential for immune evasion or neutralization. (**A**) Top view and (**B**) side view of the model of the WNV E ectodomain with the M proteins are shown. Residues Thr64 (orange), Thr208 (teal), Ala261 (red), and Leu267 (green) are shown in space-fill representation, and DENV2 Asn67 (PDB ID: 4UTC) is superposed and depicted in blue (CPK, space fill). The inset on the upper right shows the disposition of these residues as viewed perpendicular to the surface of the E dimer, and the proximity of the buried residues to the E:M interface, the latter viewed from a second perspective related by a 90° rotation, on the lower right. Source data are available online for this figure.

WNV E residues Thr208 or Thr64, contributed by chains C or E of the E protein, respectively, are positioned diagonally opposite each other near the dimer twofold axis, with the HCDR3 loop positioned in between (Fig. 3B). Both residues are surface accessible and primarily interact with residues from the respective Fab domain positioned above. Based on extensive screening of single-, double-, and triple-mutants of E protein residues in the DII domain, Goo et al identified Thr64 and Thr208 as two of six residues that led to more than a fourfold decrease in neutralization when mutated (Goo et al, 2019). Furthermore, a single point mutation of Thr208 led to a greater decrease in neutralization compared to the mutation of Thr64, and mutation at either position was critical for escape from antibody-mediated neutralization (Goo et al, 2019). Thr64 of chain E lies on strand a/b (Fig. 3B). The backbone of HCDR3 (around residues Gly107 and Ser109) is within hydrogen bond distance of the backbone of chain E residues Val65-Ser66, which lie next to Thr64. Thr64 is also surrounded by Phe92 on LCDR3 and Tyr32 of LCDR1. Acquisition of an additional N-linked glycosylation in the Thr64Asn mutant would disrupt the Fab interactions near this position, possibly directly by affecting the binding of HCDR3 in the loop, or indirectly by affecting the binding of the LC Fv domain, in turn affecting the binding of the HC Fv domain to E protein. This structure corroborates findings by Goo et al that (1) acquisition of glycosylation at Thr64 contributes to escape from recognition by this antibody and (2) regardless of glycan occupancy at this position, the neutralization potency of the Thr64Asn mutant is directly affected. The latter finding is likely due to the disruption of stabilizing interactions and the neighboring environment of L-CDR loop residues interacting with this part of the E protein. Thr208 is part of a β-turn (V^206^GT^208^) connecting strands f and g. Thr208 lies opposite to the HCDR3 lodged in the E dimer groove on one side and HCDR2 on the other. Mutations Asp67Asn, Ala261Lys, and the double mutant, Leu266Ser_Ala267Thr were shown to significantly affect neutralization potency; these residues localize to either the surface-accessible regions or are buried within the E:M tetramer (Fig. 6A,B).

## Discussion

The WNV-86 antibody is of particular interest owing to both its ultrapotent, virus- and lineage-specific neutralization and for being the first strictly DII-domain-binding WNV antibody reported to date. Here, we structurally map the WNV-86 epitope. The quaternary engagement of the discontinuous epitope involves both protomers of the dimer, which likely blocks the acid-induced conformational changes needed for membrane fusion after endocytosis-mediated virus entry. WNV-86 is the prototype of a distinct class of E-dimer epitope antibodies, which we propose here be classified as EDE class 3 (EDE3) antibodies. The salient features of EDE3 antibodies are as follows: (1) The primary binding region lies exclusively within DII and does not include any residues from DI or DIII; (2) the twofold symmetry of the E dimer is implicated in Fab binding to the dimer by engaging residues from the two protomers, which are located close to the symmetry axis; (3) the binding region of the Fab is positioned away from the 150 loop and the Fab does not interact with the glycan loop; (4) the EDE3 epitope is located far from the fusion loop and does not overlap with most of the fusion loop epitope; (5) the CDR loop of the Fab (HC Fv domain of WNV-86) is lodged in the groove formed near the E dimer twofold axis; and (6) the epitope of the EDE3 Fab overlaps with the prM binding region at two main sites (the ‘pr’ head domain and the tail region that precedes the furin cleavage site) and hence occludes WNV-86 Fab binding on virions containing prM. Combined with previous experimental data that identified residues whose mutation affected neutralization, the structure of the complex presented in this study suggests an important role for the E dimer symmetry and the integrity of the (E:M)_2_ tetramer. For instance, Ala261 localizes to helix αB close to the E protein dimer twofold axis. Similarly, by virtue of their location, a double mutant at positions 266 and 267 (to Ser or Thr, respectively) of the envelope glycoprotein, which are buried within the tetramer and lie close to the M protein, could affect the local hydrophobic environment and tetramer stability.

Positioning of the HCDR3 loop of this EDE3 antibody at the E dimer groove, as well as the potential partial overlap with prM at the β fold head domain, clarifies the selective recognition of mature particles by the WNV-86 antibody. This HCDR3 loop is approx. 5.2 Å wide, whereas the E protein dimer width is about 10.7 Å. Hence, the loop position does not require structural changes to the E protein dimer (Fig. 3B) (Hardy et al, 2021). The positioning of the HCDR3 in the dimer groove is unlikely to be a fortuitous occurrence, as evidenced by the backbone overlap of two residues with those in prM (PDB ID: 3C6E) and the proximity of HCDR3 with chain E residues, favoring potential hydrogen bond interactions (Krissinel and Henrick, 2007). The overlap of the prM binding region with EDE1 and EDE2 antibodies, which can bind partially immature virus, largely resides in the pr head domain. The EDE1 or EDE2 epitope does not overlap with the prM binding site near the E protein dimer twofold axis (Dejnirattisai et al, 2015). The cryo-EM structure of WNV complexed with the Fab of WNV-86 was reconstructed from samples prepared in buffers at pH 8.0. Previous research demonstrated that a conformational change in the low pH environment of the trans-Golgi network leads to a conformational arrangement that exposes the prM prior to its subsequent cleavage by furin (Yu et al, 2008; Zhang et al, 2004). Studies have shown that after cleavage of prM by furin, pr remains associated with E at low pH, and it is released from the virus particle of most flaviviruses only at neutral pH (Vaney et al, 2022). The structural details of the sequential conformational states accompanying the molecular changes are unclear. However, our findings show that the WNV-86 epitope would overlap with both pr and prM binding (Fig. 5) and, therefore, the antibody can only selectively bind in the absence of both pr and prM, hence WNV-86 binds strictly to the mature virus (Goo et al, 2019).

The description of the EDE3 epitope is currently mostly restricted to the WNV-86 antibody; however, this site of antibody recognition may also be present in flaviviruses from other serocomplexes. WNV-86 and ZIKV DH1017 belong to different antibody isotypes, yet the cryo-EM structure of ZIKV with bound DH1017 Fab displays an overall binding architecture similar to that observed for WNV-86. The epitopes of both ZIKV DH1017 and WNV-86 are limited to the DII domain. These differences suggest that ZIKV DH1017 may be a variation or subclass of the EDE3 antibodies. Another possible EDE3 or EDE3-like antibody may be the antibody WNV CR4348. Functional studies identified Thr208 and H246, both residing in the DII domain, as critical residues (Vogt et al, 2009). Thr208 and H246 are contributed by the opposite protomers of the dimer. CR4348 was also reported to cross-link E protomers and block the formation of the fusion-competent form of E protein, a feature shared with WNV-86 and ZIKV DH1017. To the best of our knowledge, our findings are the first to reveal the presence of EDE3 (WNV-86) and EDE3-like (ZIKV DH1017) modules that span across antibody types and valencies.

Acquisition of glycosylation in DII at Thr64 was shown to be an immune evasion strategy against the WNV-86 antibody and EDE3 antibodies (Goo et al, 2019). Indeed, ZIKV DH1017 does not bind to or neutralize DENV2, which naturally has a second glycosylation site at position 67 in DII (Singh et al, 2022). The Asn67 of DENV and Thr64 of WNV-86 both lie on the a/b strand, and the shift in the residue positions correlates with the shift in the respective Fab variable domain lying atop the residues (Fig. 4C). Surface residues Thr64 and Thr208, directly interact with Fv domains, and hence their mutation causes a significant reduction in potency. Residues buried within could be important for dimer interactions near the twofold axis (E:E or E:M) and hence the oligomer stability that may affect WNV-86 binding (Fig. 6B); however, these effects need to be ascertained experimentally. Such residues buried in the dimer interface (E:M or E:E) are strictly conserved across WNV lineages, emphasizing their possible role in stabilizing interactions for the heterocomplex. The surface residues are conserved in lineage 1 strains, underscoring the focused applicability of WNV-86 toward this lineage.

The structure of the complex of WNV E protein with WNV-86 Fab reported here also raises an important question regarding the preferential binding of the Fab to site 1 formed by envelope protein chains C and E. The quasi-twofold related binding site located on the same envelope protein dimer (chains C and E; site 2) does not show Fab density in the final reconstruction, and this site cannot support binding when site 1 is occupied, due to steric hindrance. Site 3 lies on the envelope protein chain A, which forms the dimer at the icosahedral twofold axis. Density at site 3 is sufficient evidence of Fab binding; however, it is insufficient to model the Fv domains with confidence. The final localized reconstruction reported in the study included 52.9% of all particles. Focused classification results showed that occupancies were around 28% for site 1 and about 10% for each possible symmetry-related position at site 3 (Appendix Fig. S2). The observation of differential densities and binding at the three sites of the ASU supports the non-equivalence of sites in the ASU of the mature flavivirus particle.

Comparison and analysis of the WNV structural models, with and without the WNV-86 Fab, suggest a possible basis for the preferred Fab binding at site 1. To better understand these differences, we group the epitope residues into epitope segment I and epitope segment II based on the glycoprotein chains. For site 1, segment I is defined as the epitope residues on chain C, and segment II consists of the residues of chain E. Therefore, at site 1, Thr64 of chain E and Thr208 of chain C are part of epitope segments II or I, respectively. Structural superimposition of the envelope protein chains (PDB ID: 9N3B) revealed that chains E and A, from the general or the icosahedral dimer, respectively, are similar to each other. However, chain C shows small but clear differences compared to chain E (Appendix Fig. S5A,B). Epitope segment I, for the quasi-twofold related binding site 2, is located on chain E. The differences between chains C and E localize to residues 62–68 (includes strand a), 118–123 (strand e), 205–210 (fg loop), 249–259, 272–282 (kl loop) (Fig. 3A; Appendix Fig. S5A,B). Of these structural elements, strands e and a belong to epitope segment II, whereas the fg loop and the kl loop are part of epitope segment I. In chain E, the structure corresponding to strands a and e is not recognized as β-strands by the DSSP algorithm due to disrupted mainchain hydrogen bonds. The conformations of the fg and kl loops are also different between the two chains of the dimer.

To investigate the structural differences between site 1 and site 2, we generated a new model showing the Fab bound at site 2: chain C of the new model is structurally superposed on chain E of the original model, with the rest of the protein chains and Fab riding. This showed that, on strand a, backbone and hydrogen bond distances between residues 65-66 of the envelope protein and Fv HC vary between site 1 segment II and those for site 2 segment II, due to a slight shift between the two envelope protein chains in this region. Therefore, the backbone atoms of residues in epitope segment II of site 2 would clash with the HCDR3 loop of the Fab, which makes site 2 inherently less favorable for binding (Appendix Table S2). Additional clashes are present with side chain atoms at site 2 (Appendix Table S2). All the clashes with the HC Fv domain residues involve epitope segment II. These distances are comparable for sites 1 and 3. In each case, clashes are most severe at site 2 and intermediate at site 3, which correlates with differences in the Fab binding and densities at the corresponding sites. Therefore, of the three sites, site 1 is most favorable for binding; site 2 has significant clashes to abrogate /eliminate binding completely, and site 3 has fewer clashes and could support binding better than site 2.

Some limitations of the study are the data, which were limited both in the number of particles and the physical pixel size chosen for data collection at the time, and the complex generated with Fabs. To estimate the feasibility of IgG binding to dimers in the raft, we measured the distances between the nearest Cα atoms of Fv HC domain C-terminal residue 124. The distance between these atoms of Fabs attached to two neighboring sites 1 (at the icosahedral fivefold axis) is 96.8 Å; between site 1 and site 3, this distance was 55.8 Å, and between two neighboring sites 3 (belonging to different envelope protein dimers) is 162.3 Å. The structure and flexibility of antibodies, including IgG1, probed using several methods, including those that account for individual antibody molecules, revealed the inherent wide flexibility and dynamics of IgG molecules (Huhn et al, 2025; Preiner et al, 2014; Roux, 1999; Saphire et al, 2002; Sosnick et al, 1992; Zhang et al, 2015). Specifically, the Fab-Fab peak distance in IgG1 molecules is ~82 Å, with distance in a potentially extended conformation up to ~170 Å (Preiner et al, 2014; Saphire et al, 2002; Sosnick et al, 1992). Therefore, the two Fabs of the WNV-86 IgG antibody can potentially bind to any two adjacent sites 1–3, barring simultaneous binding to the twofold and quasi-twofold sites subject to steric hindrance. The WNV-86 antibody could also connect site 1 and site 3, and hence the dimers within the raft. However, the dynamics of binding of the two Fab arms, at sites 1–3, could be non-identical (Natesan and Agrawal, 2023). The reported complex was generated at 37 °C, and differences in occupancy with temperatures were not investigated as part of this study.

All E dimer-binding IgG antibodies and the ZIKV DH1017 IgM are ultrapotent (IC_50_ less than 10 ng/ml); however, not all ultrapotent antibodies bind E dimers (Dejnirattisai et al, 2015; Fibriansah et al, 2025; Rouvinski et al, 2015; Shu et al, 2025). Ultrapotent E dimer-binding antibodies share several features. For instance, the EDE1 and EDE2 antibodies bind quaternary epitopes involving the highly conserved fusion loop, and therefore, not surprisingly, these are also cross-reactive (Barba-Spaeth et al, 2016). Further, these ultrapotent E dimer-binding antibodies cross-link the E protomers, including WNV-86; this likely interferes with the conformational changes required for membrane fusion, a precursor to elicit and sustain viral infection (Crowe, 2017; Tortorici et al, 2020). Yet, of all these antibodies, WNV-86 is the most potent at an IC_50_ of 2 ng/ml, suggesting that structural elements dictating the exceptional potency of WNV-86 lie beyond the generalized shared features (Goo et al, 2019). Differences between EDE1-3 lie in the details of their epitopes and the nature and site of cross-linking. The WNV-86 epitope is highly specific, both for the virus and WNV lineage, involves a minimalist DII quaternary epitope, and an HCDR3 uniquely positioned at a site not observed for any previous flavivirus antibody. All these WNV-86 features may contribute to its exceptional potency. The site of the HCDR3 insertion could be especially pertinent if the instant center of rotation during any conformational changes lies near the site of loop insertion. Similar structural correlations between the antigenic site and the site of conformational transition that affect potency have been reported for other viruses (Banerjee et al, 2022; Crowe, 2017). While molecular mimicry was not detected between HCDR3 and prM, the role of the residues and sequence of the HCDR3 loop in the specific positioning at the dimer interface and the antibody potency merits investigation. Bivalent binding and multi-site engagement (as discussed above), which are not present in the reported structure, may be feasible in solution and could expand the range of binding or potency. Further studies are needed to resolve these questions and details of the epitopes of EDE3 antibodies at the general and icosahedral sites. Such work also might help us to better understand the basis of differences between IgG and IgM antibodies in disease protection or enhancement. Harnessing these insights may allow a broader applicability of using EDE1-3 antibodies as medical countermeasures to mitigate the burden of flavivirus disease.

## Methods

**Table:** Reagents and tools table

Reagent/resource	Reference or source	Identifier or catalog number
**Experimental models**		
N/A		
**Recombinant DNA**		
N/A		
**Antibodies**		
WNV-86 IgG antibody	Crowe Lab, Vanderbilt University Medical Center	–
**Oligonucleotides and other sequence-based reagents**		
N/A		
**Chemicals, enzymes, and other reagents**		
HEPES	Millipore Sigma	Cat# H3375-500G
Gibco Fetal Bovine Serum	Millipore Sigma	Cat# F0926-500
Tris buffer	Fisher Scientific	Cat# BP152-1
Sodium chloride	Fisher Scientific	Cat# S271-3
PEG8000	Sigma-Aldrich	Cat# P2139
Sucrose	Sigma-Aldrich	Cat# S9378
Glycerol	Fisher Scientific	Cat# G31-1
Potassium tartrate	Sigma	Cat# 25510-500 G
**Software**		
Leginon	https://github.com/leginon-org/leginon	–
Appion	http://www.appion.org	–
CryoSPARC	https://cryosparc.com/	–
ChimeraX	https://www.cgl.ucsf.edu/chimerax/	–
Chimera	https://www.rbvi.ucsf.edu/chimera/	–
MolProbity	https://molprobity.biochem.duke.edu	–
DAQ score	Kiharalab Cryo-EM Suite: https://kiharalab.org/emsuites/daq.php	–
Coot	https://www2.mrc-lmb.cam.ac.uk/personal/pemsley/coot/	–
Phenix	https://www.phenix-online.org/	–
PISA server	https://www.ebi.ac.uk/pdbe/pisa/	–
PyMOL	https://www.pymol.org/	–
ESPript	https://espript.ibcp.fr/ESPript/ESPript/	–
T-Coffee	https://www.ebi.ac.uk/jdispatcher/msa/tcoffee	–
**Other**		
C6/36 cells	ATCC	CRL-1660
WNV Kunjin virus strain MRM61C	Kuhn Lab, Purdue University	–
Pierce Fab preparation kit	Thermo Fisher Scientific	Cat# 44985
Gibco Trypsin-EDTA	Thermo Fisher Scientific	Cat# 25-200-056
Gibco Minimal Essential Medium	Thermo Fisher Scientific	Cat# 11-095-080
Amicon Ultracentrifugal filters (100 kDa MWCO)	Millipore	Cat# UFC810024
Ultrathin lacey carbon grids	Ted Pella Inc.	Cat# 01824

### Fab production

Production and characterization of the WNV-86 antibody were described previously (Goo et al, 2019). WNV-86 Fab fragments were generated from purified antibody using a Pierce^TM^ Fab Preparation Kit (Thermo Fisher Scientific). Briefly, antibodies at a starting concentration of 1 mg/mL were used for incubation with immobilized papain at 37 °C for a duration of 3 h. The Fab fragments produced were then purified from the digested solution by passage through a protein A column (Nab Protein A Plus Spin Column, Thermo Fisher Scientific). Purified Fab in PBS was then concentrated to approximately 10 mg/mL, as determined using a NanoDrop spectrophotometer (Thermo Fisher Scientific) and stored at −20 °C.

### Fab-virus complex generation

WNV was propagated and purified as described previously (Therkelsen et al, 2018). Briefly, C6/36 cells were grown at 30 °C using Gibco Minimal Essential Medium (Thermo Fisher Scientific) supplemented with 1× nonessential amino acids, 20 mM HEPES pH 7.3, and 10% FBS. Cells were inoculated with KUNV virus (strain MRM61C) at a multiplicity of infection of 1, and virus-containing medium was harvested 50- and 65-h post-infection. The medium was clarified by centrifugation for 20 min at 8000 rpm (rotor JA-10, Beckman Avanti) at 4 °C. PEG8000 was added to the collected supernatant to a final concentration of 8% and incubated overnight with stirring at 4 °C. The medium was then centrifuged for 30 min at 8000 rpm (JA-10 rotor, Beckman Avanti) at 4 °C. Pellets were resuspended in TNE (10 mM Tris pH 8, 120 mM NaCl, 1 mM EDTA) and ultracentrifuged over a 22% (w/v) sucrose solution for 2 h at 32,000 rpm (Ti50.2 rotor, Beckman Coulter, Inc.) at 4 °C. Unclear pellets were resuspended in TNE and centrifuged for 20 min at 5800 rpm at 4 °C (rotor JA-10, Beckman Avanti). Clear, resuspended pellets were ultracentrifuged over a discontinuous glycerol-tartrate gradient (positive density gradient of potassium tartrate ranging from 0 to 50% (w/w) in 10% increments and negative viscosity gradient of glycerol from 35% to 10% in 5% increments) for 2 h at 32,000 rpm (SW41 rotor, Beckman Coulter, Inc.) at 4 °C (Ashley and Caul, 1982). Virus bands were visualized using blue light near the 20–25% gradient interface. The volume corresponding to the bands was recovered, and the purified virus was buffer exchanged in TNE and concentrated using a 100 kDa molecular cut-off ultracentrifugal filter (Millipore) to a final volume of ~50 μL. Purity, concentration, and the presence of immature virus particles were assessed through SDS PAGE with BSA standards. Fab-virus complexes were generated by overnight incubation at 4 °C.

### Cryo-EM data collection

Ultrathin lacey carbon grids (400 mesh, Ted Pella Inc.) were used for cryo-EM data collection. A volume of 2.5 µL of the Fab-virus complex was added to the carbon side of non-glow discharged grids at ~85% humidity, and the grids were then plunge-frozen into liquid ethane using a CP3 plunger (Gatan) within a BSL2-level certified biosafety cabinet. Grids of vitrified virus-Fab samples were screened using a Talos F200C (Thermo Fisher Scientific) operated at 200 kV and equipped with a Ceta 16 M camera (Thermo Fisher Scientific). After optimization of the cryo-EM sample preparation conditions, frozen grids with vitrified Fab-virus complex were used to collect data for single particle cryo-EM data. Approximately 1117 micrographs were collected using a Titan Krios G1 microscope (FEI) operated at 300 kV and equipped with a K2 Summit direct electron detector (Gatan), using Leginon (Carragher et al, 2000). Data were collected at a magnification of ×81,000 in counting mode, and hence a pixel size of 1.73 Å. Frames were recorded every 0.2 s for 8 s, with a dose rate of ∼8 electrons per physical pixel per second. The total dose for each image stack of 40 frames was ∼24 electrons per Å^2^. The nominal defocus range used was −0.9 to −2.0 µm.

### Data processing and model building

Single-particle frames were processed for motion correction and CTF estimation using Appion (Lander et al, 2009), and the downstream processing of the dose-weighted frames was performed using CryoSPARC version 4.5.3 (Punjani et al, 2017). Initially, 30,901 particles were picked and extracted from 1117 micrographs based on an estimated diameter of an antibody-bound flavivirus particle. After four iterations of 2D classification steps in CryoSPARC, 10,275 particles were used to generate a template. The template picker module was used to re-extract 190,310 particles. A single class generated by the subsequent 2D classification, consisting of 12,760 particles, was used for ab initio model generation and subsequent 3D reconstruction and homogenous refinement. The gold-standard FSC curve using two half-maps and a resolution cut-off at 0.143 was used to determine the resolution of the 3D reconstruction. For the localized reconstruction, first, the center of an asymmetric unit was defined by fitting a model of the glycoproteins and Fab in the icosahedrally averaged map (ChimeraX) and then symmetry expanded to create 60 copies. Each copy was extracted from the original micrographs and reconstructed into a consensus 3D model, which was then subject to 3D classification. About 52.9% of all particles showed strong density for the Fabs and were subsequently refined to a resolution of 3.9 Å. Further classification with focused masks for the three sites individually showed occupancy of 28.1% for site 1, about 10% for both potential binding modes on site 3, and no occupancy for site 2. Further refinement for the particles in these classifications did not improve the resolution or density of the maps, likely because of the limited number of particles. All steps were done in CryoSPARC.

Model building was performed in Coot (Emsley and Cowtan, 2004). An initial model for the three E:M heterodimers was constructed using the poly-Ala backbone generated from the Usutu SAAR-1776 (PDB ID: 7LCH) model, since this represents the highest resolution reconstruction for mature flavivirus and hence has low errors in peptide geometry. A model for WNV-86 was generated from the crystal structure model of the Fab of WNV E53 antibody (PDB ID: 3I50). The model of the glycoprotein complex with WNV-86, corresponding to the asymmetric subunit of the icosahedron, was fitted into the 3D reconstruction using Chimera (Pettersen et al, 2004) and was refined in real space with the phenix.refine module within Phenix (Adams et al, 2010). Model geometry and related parameters were assessed using Molprobity and DAQ-score (Terashi et al, 2022). Manual corrections of model errors, real-space refinement, and model assessment were iteratively performed using Coot and Phenix. Structural analyses were performed using the PISA server, PyMOL, Chimera, and ChimeraX for graphics and ESPript (https://espript.ibcp.fr) and T-Coffee for multiple sequence alignments (Emsley and Cowtan, 2004; Gouet et al, 2003; Krissinel and Henrick, 2007; Pettersen et al, 2004).

## Supplementary information

Appendix

Peer Review File

Source data Fig. 1

Source data Fig. 2

Source data Fig. 3

Source data Fig. 4

Source data Fig. 5

Source data Fig. 6

Figure EV1 Source Data

Figure EV2 Source Data

Figure EV3 Source Data

Expanded View Figures

## Data Availability

The cryo-EM map and the associated model were deposited to the Electron Microscopy Databank (EMDB) or the Protein Databank (PDB), respectively, with the following EMDB and PDB IDs: EMD-48850 and 9N3B. The source data of this paper are collected in the following database record: biostudies:S-SCDT-10_1038-S44319-026-00851-z.
