# Supplementary material for: A distinct E dimer epitope underlies selective recognition by a protective human West Nile virus antibody
Source: EMBO Rep. 2026 Jun 29;27(15):4583–601. doi: 10.1038/s44319-026-00851-z (PMC13458482; doi:10.1038/s44319-026-00851-z)
Supplement: Supplementary file 1 [file 44319_2026_851_MOESM12_ESM.pdf]

Expanded View Figures

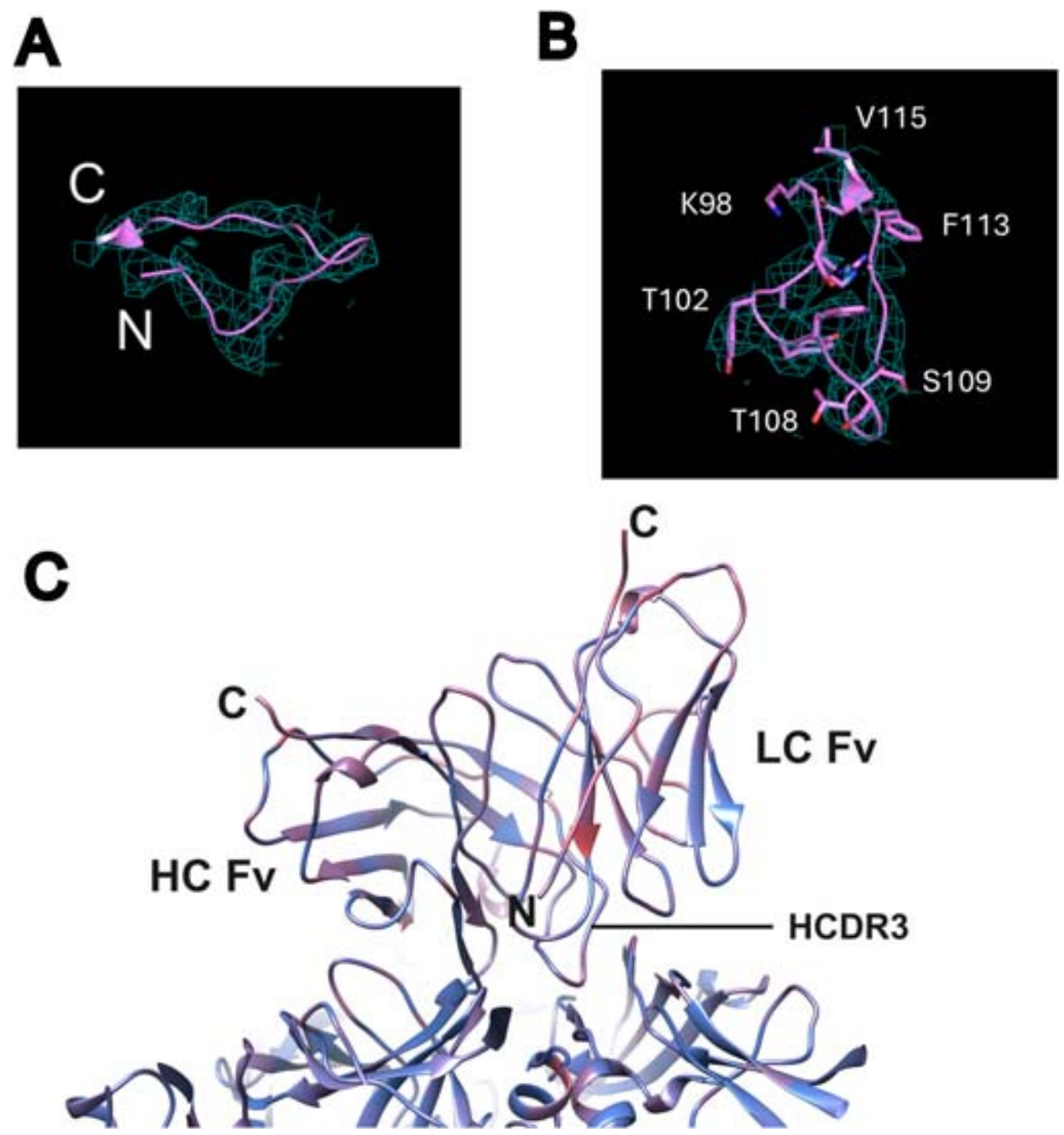

Figure EV1. Density for the HCDR3 loop and model-to-map quality.

(**A**) The density around the HCDR3 loop is shown with the loop represented in cartoon (sigma 1.0). (**B**) Side chains of some of the residues within the loop are depicted in sticks and labeled. The side chain density for bulky side chains corroborates a reasonable fit of both residues and the loop in the cryoEM map. (**C**) The Q score indicates the resolvability of the atoms in the map. An overall score of 0.52 and the model indicate that the residues and side chains are well resolved. Source data are available online for this figure.

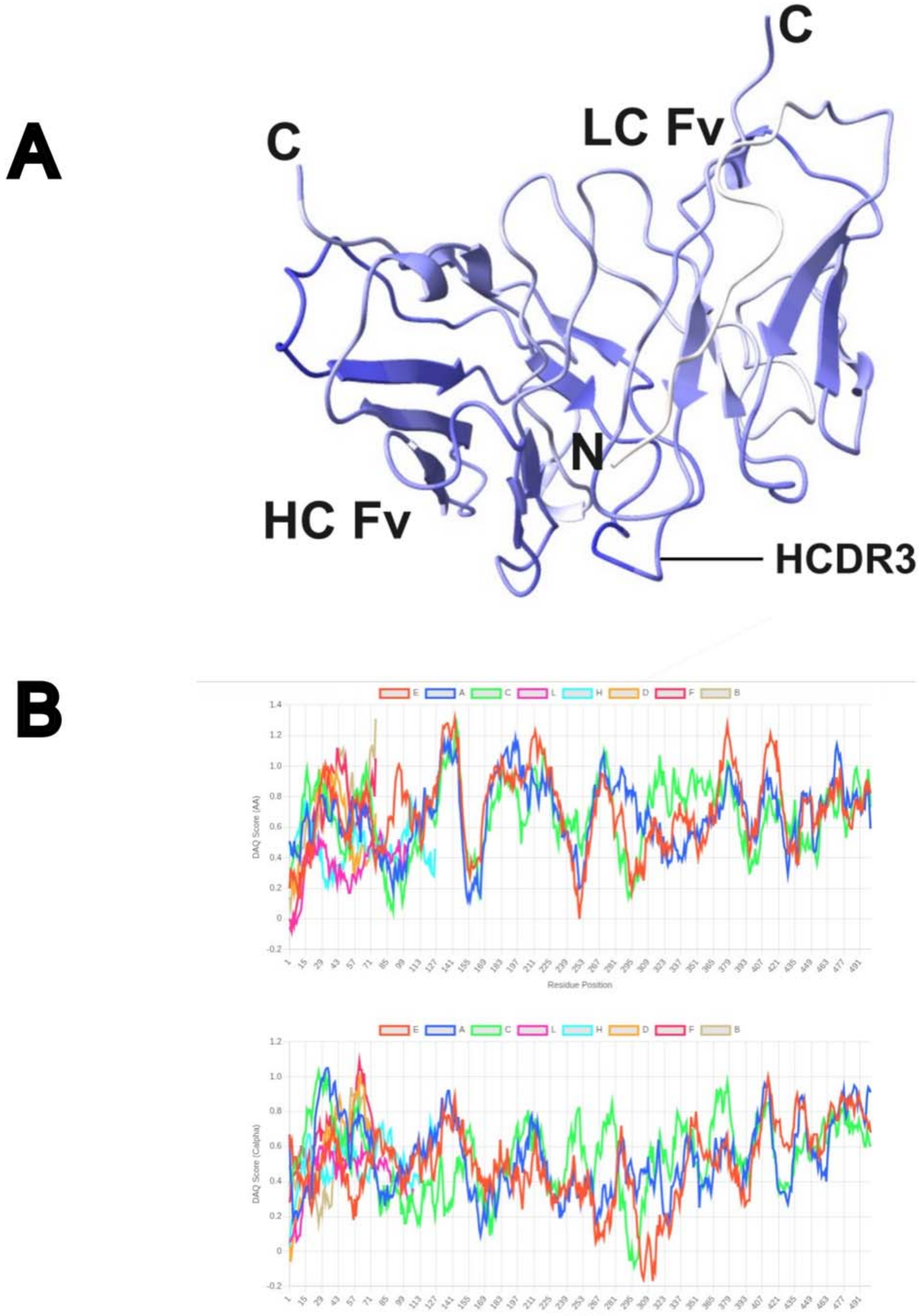
A
C
LC Fv
C
N
HC Fv
HCDR3
B
E A C L H D F B
DAQ Score (AA)
Residue Position
E A C L H D F B
DAQ Score (Calpha)
Residue Position

Figure EV2. The DAQ score of WNV-86 Fab.

(**A**) The DAQ score quantifies the local quality of the model and can identify wrongly modeled residues and regions. The score is from −1 (red), 0 (white) to +1 (blue). The DAQ score on the model of the E and Fab complex shows a reasonably accurate fit and quality for both E and the Fab, and especially for the HCDR3 loop region. (**B**) The graph shows the DAQ score by residues in different chains in the complex. The positive values for both Fv domains indicate a reasonably accurate fit of the Fab model in the cryoEM map. Source data are available online for this figure.

© The Author(s)

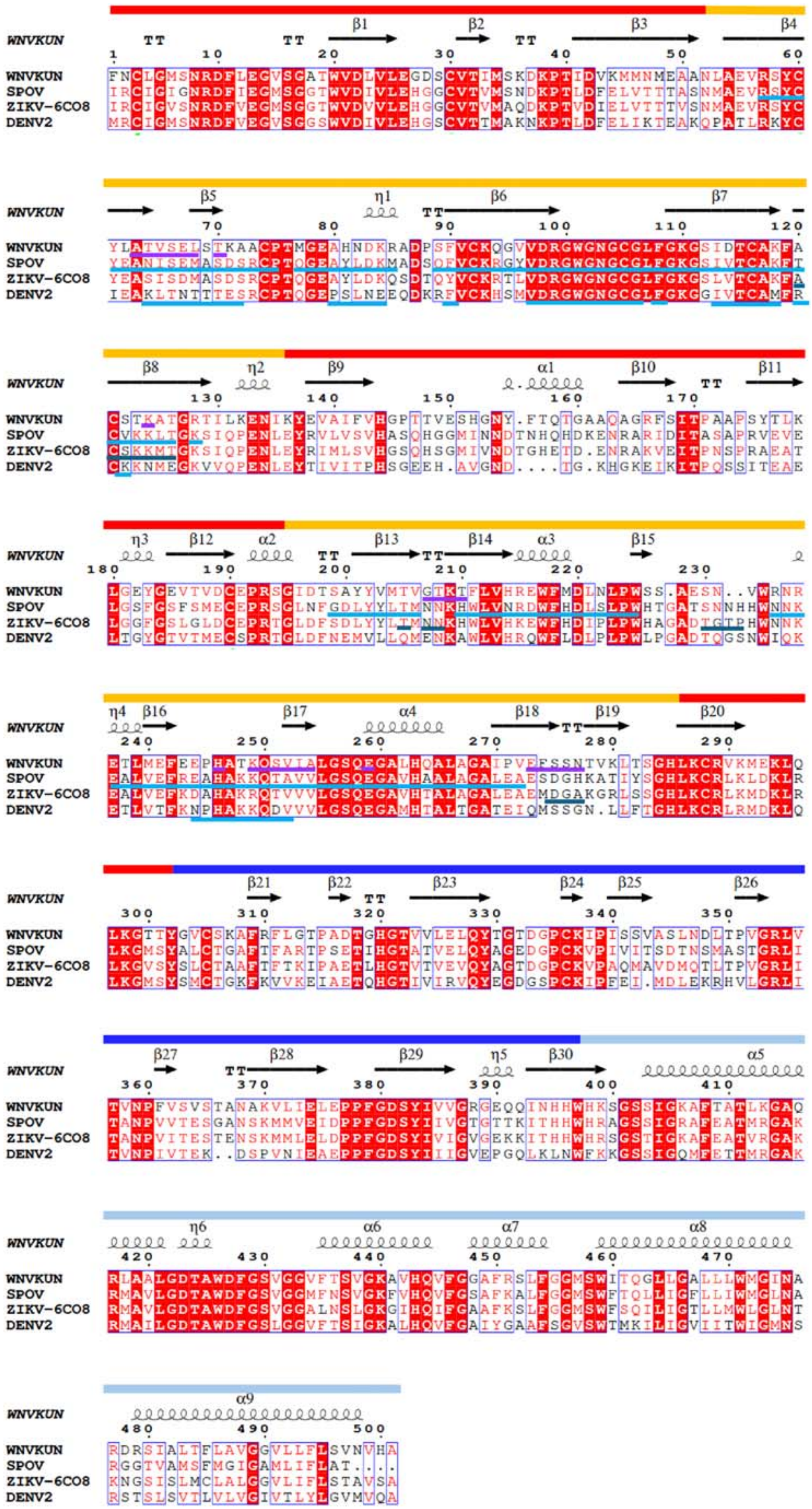
WNVKUN
SPOV
ZIKV-6CO8
DENV2

Figure EV3. Interface residues mapped on E sequences.

Sequences of E proteins from WNV, SPOV, ZIKV, and DENV2 are aligned using ESPript 3.0. Interface residues (within 4.5 Å contact distance) of WNV with WNV-86 are underlined in purple. The E residues for recombinant pr (DENV2, PDB code: 3C6E) and prM (SPOV, PDB code: 6ZQI) are underlined in cyan. The contact residues in ZIKV E for ZIKV DH1017 are underlined in teal. A colored bar above the sequences represents domains I-III (red, yellow, or blue, respectively) for WNV E protein. Source data are available online for this figure.
