# Supplementary material for: A distinct E dimer epitope underlies selective recognition by a protective human West Nile virus antibody
Source: EMBO Rep. 2026 Jun 29;27(15):4583–601. doi: 10.1038/s44319-026-00851-z (PMC13458482; doi:10.1038/s44319-026-00851-z)
Supplement: Supplementary file 2 [file 44319_2026_851_MOESM1_ESM.pdf]

Appendix to

A distinct E dimer epitope underlies selective recognition by a protective human West Nile virus antibody

Baldeep Khare†, Charles-Adrien Arnaud†, Thomas Klose, James E. Crowe, Jr. and Richard J. Kuhn*

*Correspondence: kuhnr@purdue.edu (R.J.K.)

†These authors contributed equally to this work.

List of Appendix Figures and Tables:

Appendix Figure S1…………………………..page 2

Appendix Figure S2…………………………..page 4

Appendix Figure S3…………………………..page 5

Appendix Figure S4…………………………..page 6

Appendix Figure S5…………………………..page 9

Appendix Table S1…...………………………page 10

Appendix Table S2……..……………………page 11

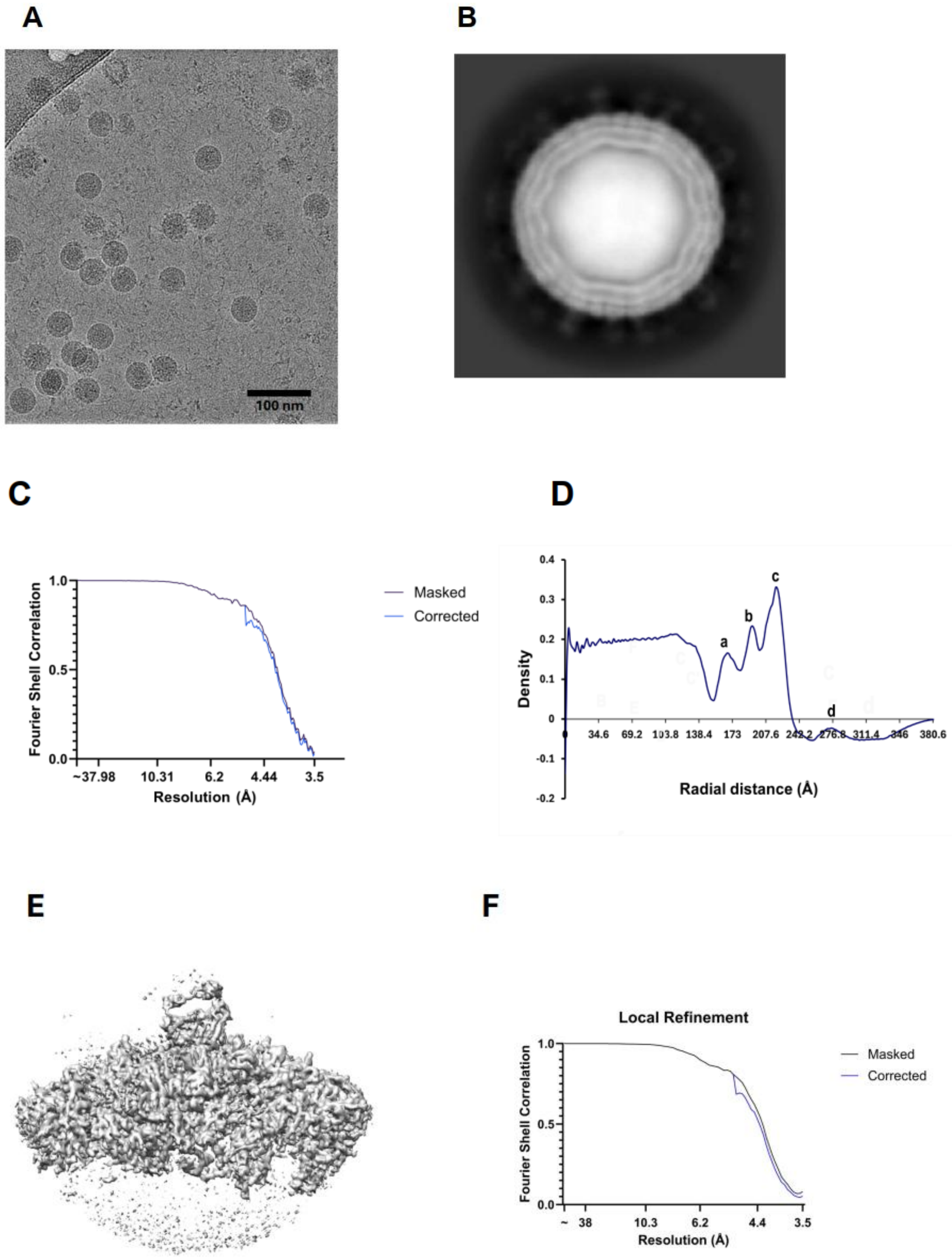
A
100 nm
B
C
Fourier Shell Correlation
1.0
0.5
0.0
~37.98
10.31
6.2
4.44
3.5
Resolution (Å)
Masked
Corrected
D
Density
0.4
0.3
0.2
0.1
0
-0.1
-0.2
34.6
69.2
103.8
138.4
173
207.6
242.2
276.8
311.4
346
380.6
a
b
c
d
Radial distance (Å)
E
F
Local Refinement
Fourier Shell Correlation
1.0
0.5
0.0
~ 38
10.3
6.2
4.4
3.5
Resolution (Å)
Masked
Corrected

Appendix Figure S1. Cryo-EM data collection and processing.

(**A**) A representative micrograph of WNV with bound WNV-86 Fab as observed with the Titan Krios G1 microscope (nominal magnification, 81,000x). (**B**) 2D classification of extracted Fab-bound WNV particles, showing an average of 12,760 particles. (**C**) An FSC plot of the map of the full particle shows the resolution at a cutoff of 0.143. The x and y axes represent the coulomb potential resolution (Å) and the correlation coefficient, respectively. (**D**) The radial density distribution plot shows peaks corresponding to the inner leaflet (a), the transmembrane domain embedded in the outer leaflet (b), the E ectodomain (c) and the WNV-86 Fab (d). (**E**) Map of localized reconstruction of particles and (**F**) the FSC curve for the localized reconstruction.

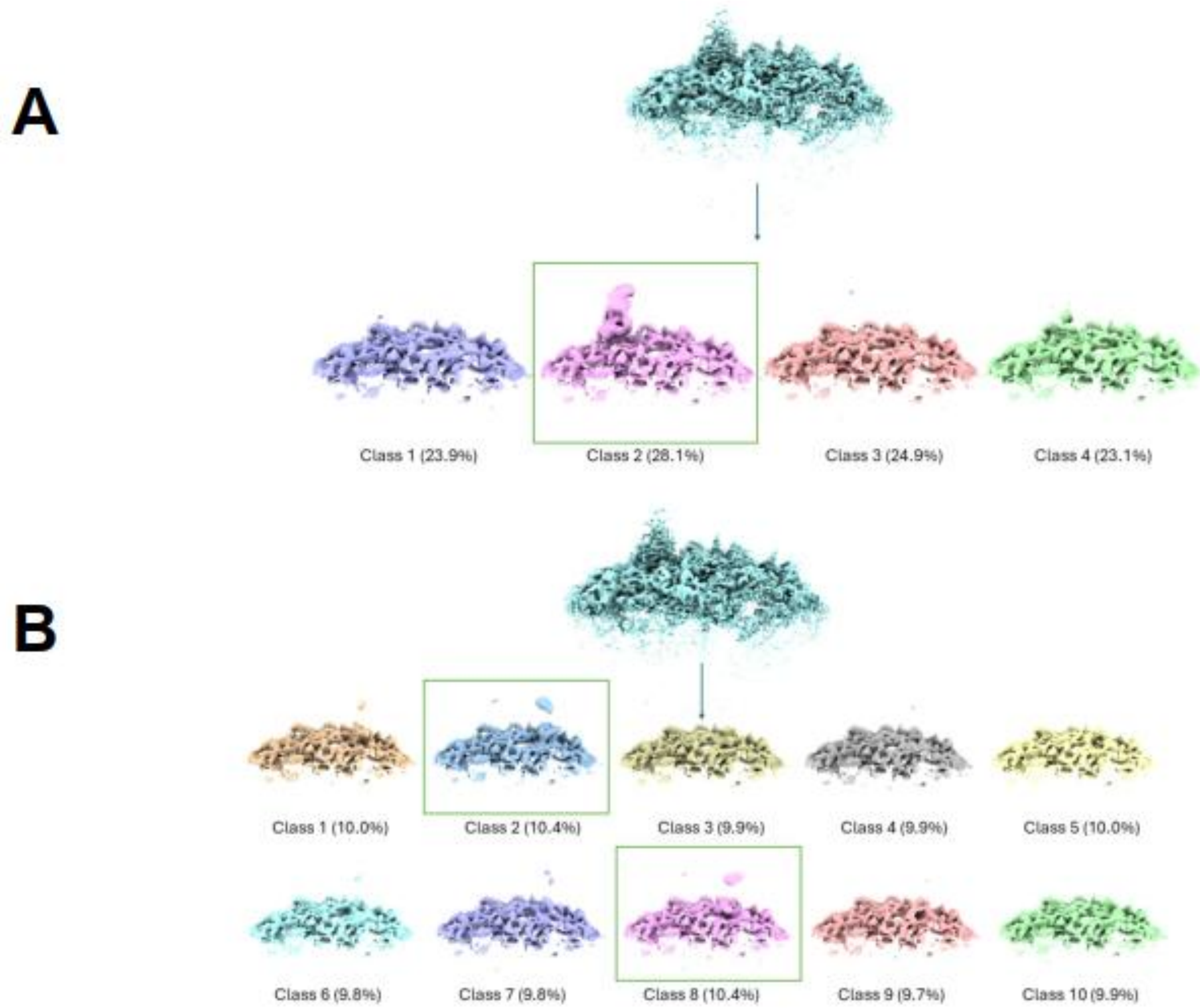

Appendix Figure S2. (**A**) 3D classification of the Fab bound at site 1

The map originating from the localized reconstruction of all particles (density in light blue) was used to create a mask around the Fab density at site 1. 3D classification with this focus mask showed that about 28% of all asymmetric units had Fab density at this site (green box).

(**B**) 3D classification of the Fab bound at site 3

The map originating from the localized reconstruction of all particles (density in light blue) was used to create a mask around the Fab density at site 3. 3D classification with this focus mask showed about 10% occupancy each for both potential binding modes on site 3 (green boxes).

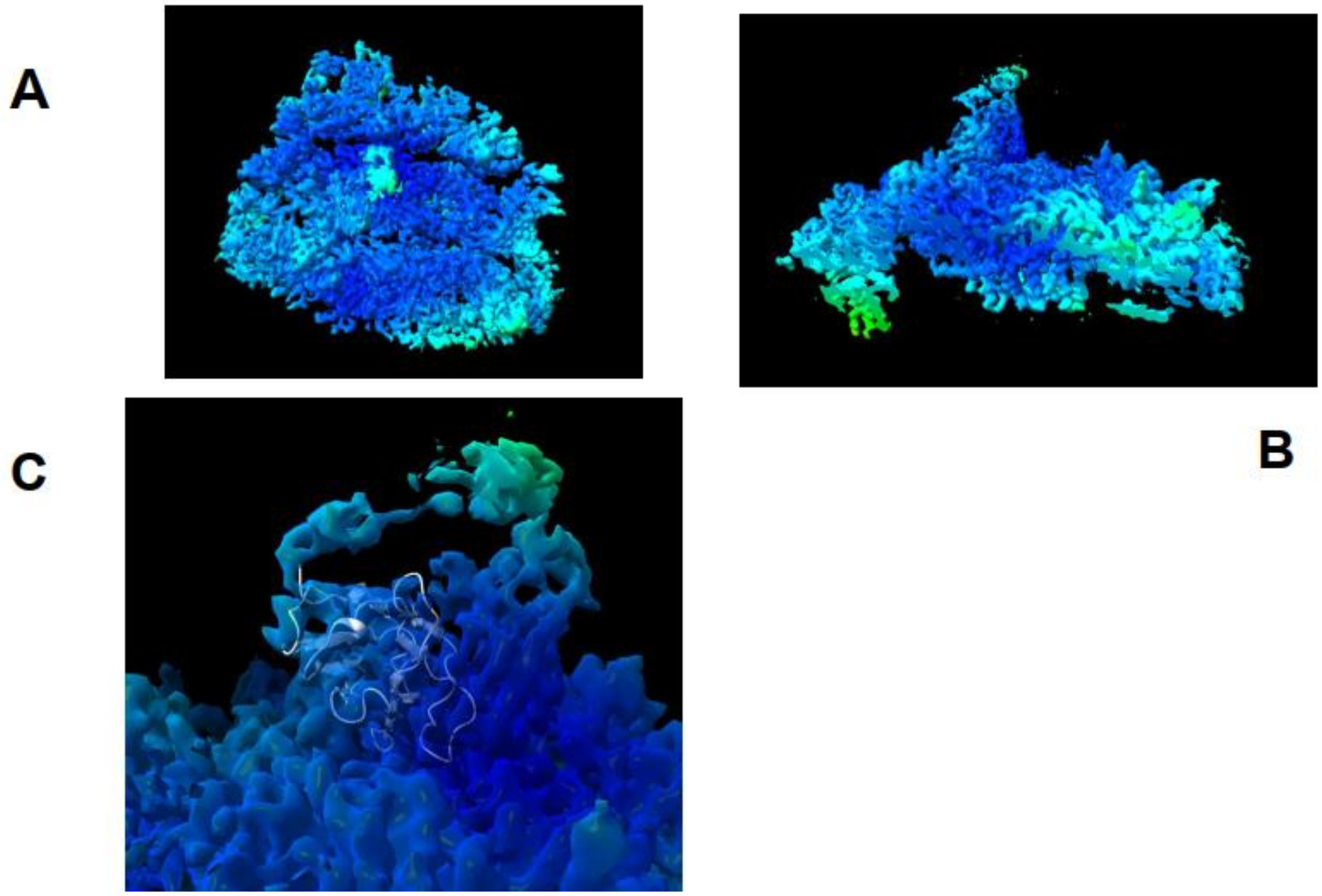

Appendix Figure S3. Local resolution.

The localized reconstruction is viewed from different perspectives (**A**) and (**B**) and mapped according to local resolution (3.6Å colored as dark blue, up to ~6Å in cyan). (**C**) The model of the Fab heavy chain Fv and the resolution at the HCDR3 loop is shown.

A

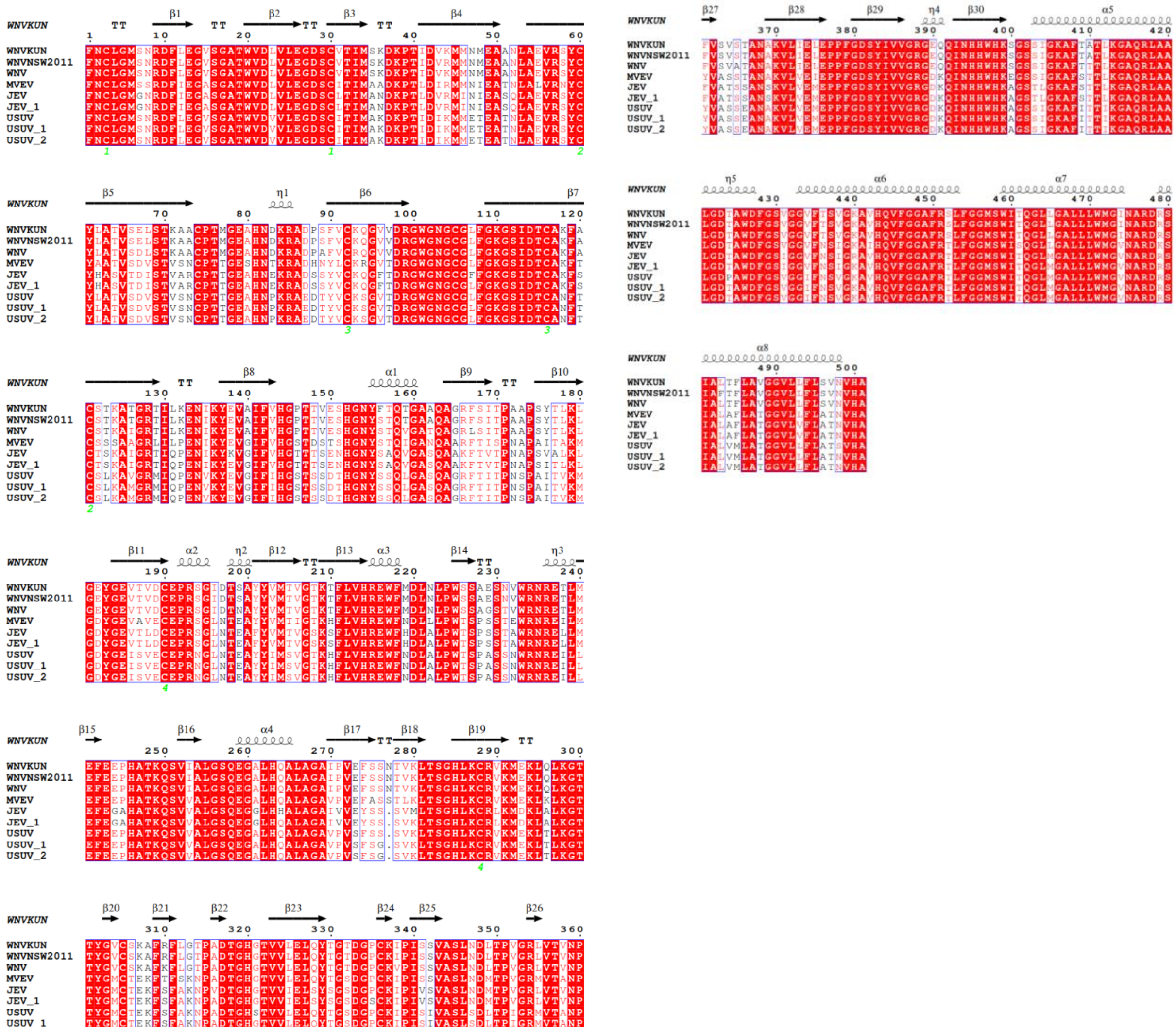

B

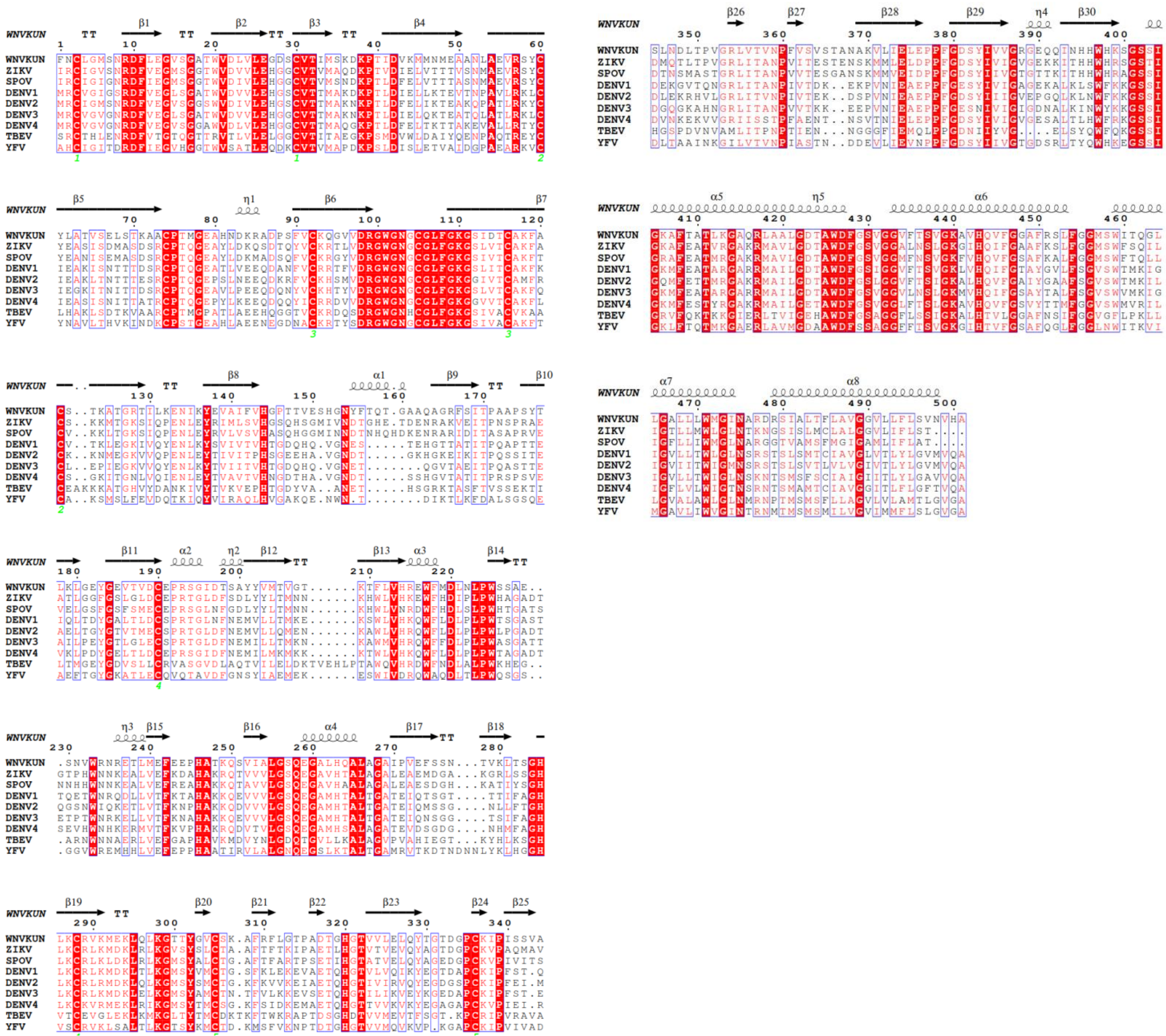

C

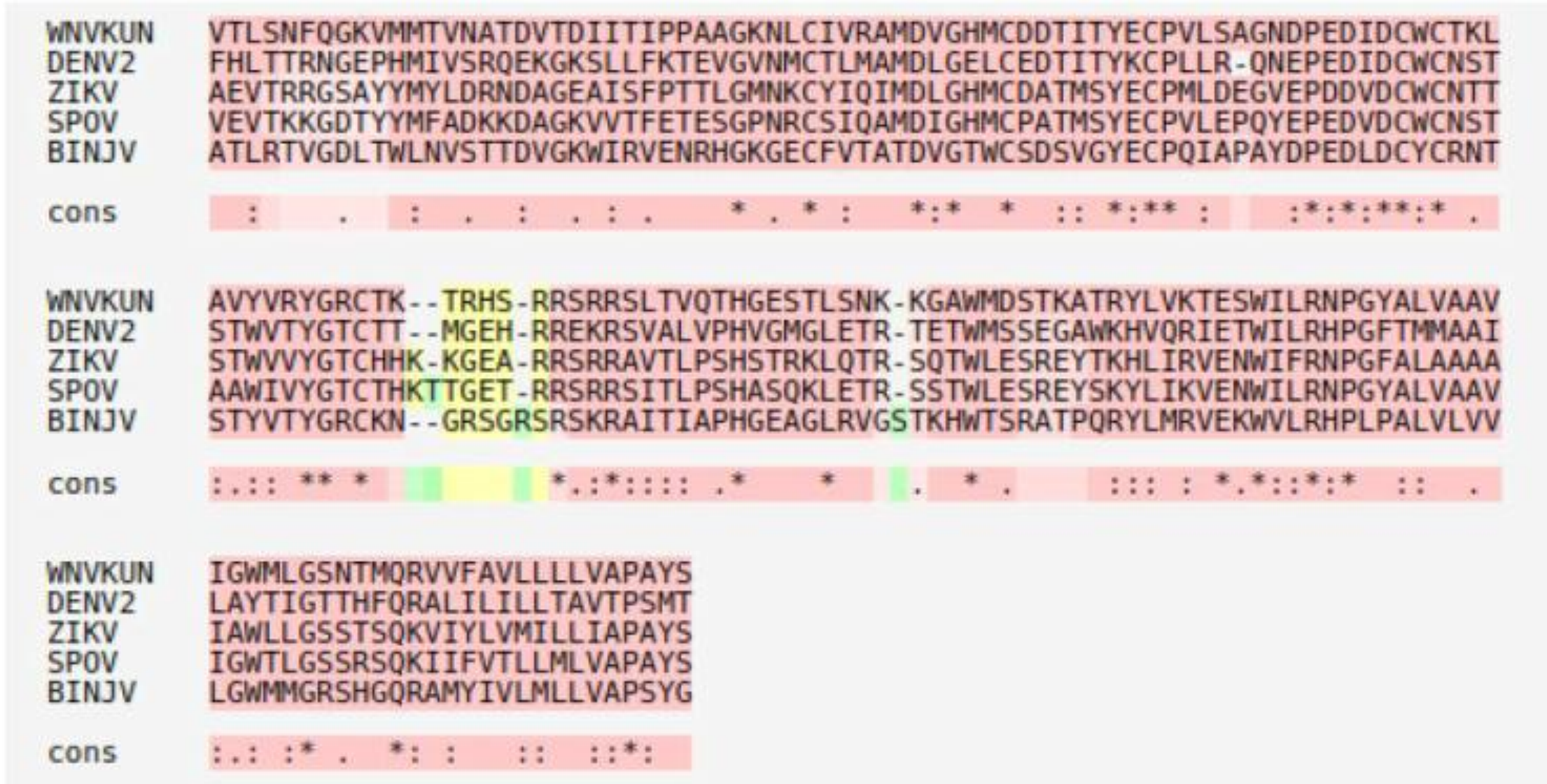

Appendix Figure S4. Multiple sequence alignments.

Sequence alignment of (**A**) E proteins from different flaviviruses belonging to the Japanese Encephalitis virus serocomplex. Sequences are aligned using ESPripT 3.0. (**B**) The E proteins from flaviviruses from several serogroups. Sequences are aligned using ESPripT 3.0. (**C**) The prM proteins of select flaviviruses. Sequences are aligned using T-Coffee.

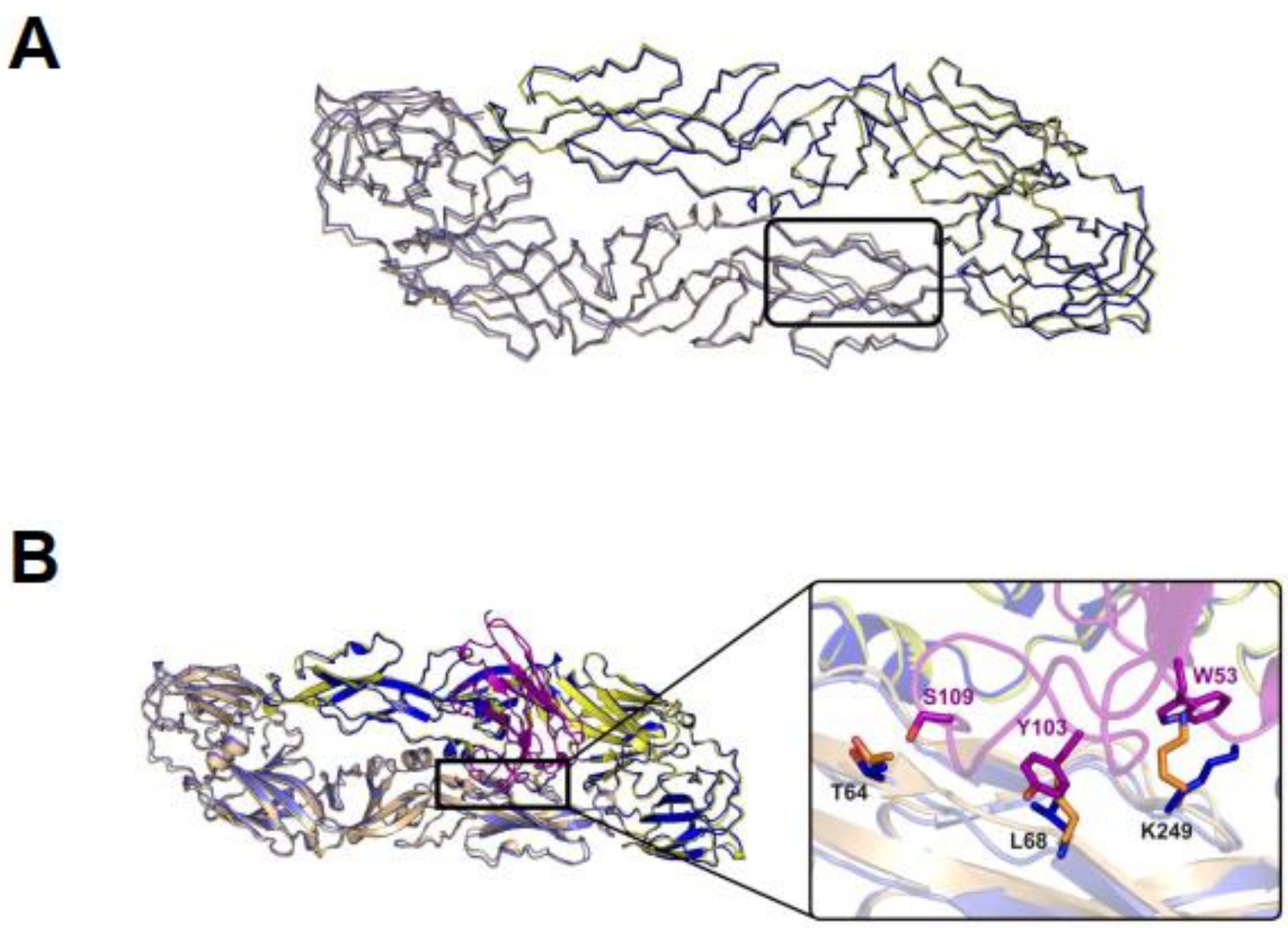

Appendix Figure S5. Close contacts and clashes at Site 2.

(**A**) Superposition of the dimer on itself with a 180º rotation, specifically with chain C superimposed on chain E, is shown. This generates the second site in the ASU, site 2 segment II (black box) where the two-fold related Fab would bind. The black box highlights the β strand shift at site 2, compared to site 1. For the original dimer, chains C and E are shown in brown or yellow, respectively. The corresponding superimposed chains are in purple or blue, respectively. (**B**) A cartoon representation of the dimers with clashes at three pairs of residues are shown. The inset shows the β strands and the residues (from site 2, segment II), which would be involved in main and side chain clashes with the Fab.

Appendix Table S1. Cryo-EM data collection, data processing and model validation statistics.

Data collection and processing	
Magnification	81,000
Voltage (kV)	300
Microscope	Titan Krios G1 (FEI)
Detector	K2 (Gatan)
Pixel size (Å)	1.73
Readout mode	Counting
Defocus range (µm)	-0.9-2.0
Total dose (e-/Å^2)	24
Total exposure (s)	8
Dose rate (e-/Å^2/s)	8
Symmetry imposed	*I*
Number of micrographs	1117
Frames per micrograph	40
Number of particles extracted	30,901
Percent of particles for used for 2D classification	37
Number of particles used for reconstruction	12760
Map resolution (Å)	3.8
Initial model used (PDB ID)	7LCH
PDB ID	9N3B
EMDB ID	EMD-48850
Refinement	
Resolution (Å)	3.8
Mask CC	0.82
Volume CC	0.83
Average B factor (Å^2)	131.69
R.m.s. deviations	
Bond lengths (Å)	0.004
Bond angles (º)	0.469
Validation	
MolProbity score	2.09
Clashscore	8.7
Poor rotamers (%)	2.46
Ramachandran plot	
Favored (%)	95.26
Outliers (%)	0

Appendix Table S2. Distances between pairs of residue atoms between the Fab and the envelope glycoprotein at sites 1-3.

Rows in bold indicate clashes involving Cα or Cβ atoms of residues.

Glycoprotein - Fab residues/ atoms	Epitope Segment	Site 1 (Å)	Site 2 (Å)	Site 3 (Å)
Thr64 Cγ2, HC Ser109 Oγ	II	3.2	2.2	4.8
**Val65 CO HC Ser109 Cα**	**II**	**3.2**	**2.1**	**4.3**
Val65 Cγ2, HC Thr108 Oγ1	II	5.3	2.4	5.3
**Ser66 Cα HC Gly110 Cα**	**II**	**3.6**	**2.4**	**3.7**
**Leu68 Cδ1, HC Tyr103 Cβ**	**II**	**3.6**	**1.5**	**2.2**
Lys249 ε-amine, HC Trp53 (Cγ, Cε2)	II	4.6, 3.6	1.0 – 1.9	N/A
Ser251 Oγ HC His57 Nε2	II	3.1	2.3	3.9
Ile253 Cδ1, HC Gly105 mainchain O	II	4.1	2.5	4.1
Ser66 Oγ, LC Tyr32 Cζ	II	3.7	2.4	3.8
Ser122 Oγ LC Phe92 Cζ	II	3.6	2.1	1.9
