# Supplementary material for: A distinct E dimer epitope underlies selective recognition by a protective human West Nile virus antibody
Source: EMBO Rep. 2026 Jun 29;27(15):4583–601. doi: 10.1038/s44319-026-00851-z (PMC13458482; doi:10.1038/s44319-026-00851-z)
Supplement: Supplementary file 3 [file 44319_2026_851_MOESM2_ESM.pdf]

EMBO reports

A distinct E dimer epitope underlies selective recognition by a protective human West Nile virus antibody

Baldeep Khare, Charles Arnaud, Thomas Klose, James Crowe, and Richard J. Kuhn

Corresponding author(s): Richard J. Kuhn (kuhnr@purdue.edu)

Review Timeline:

Transfer Date:	15th Dec 25
Editorial Decision:	17th Dec 25
Revision Received:	28th Apr 26
Editorial Decision:	21st May 26
Revision Received:	11th Jun 26
Accepted:	17th Jun 26

Editor: Achim Breiling

Transaction Report: This manuscript was transferred to EMBO Reports following peer review at The EMBO Journal.

(Note: With the exception of the correction of typographical or spelling errors that could be a source of ambiguity, letters and reports are not edited. Depending on transfer agreements, referee reports obtained elsewhere may or may not be included in this compilation. Referee reports are anonymous unless the Referee chooses to sign their reports.)

Dear Dr. Kuhn,

Thank you for transferring your manuscript to EMBO reports. I now went again through the manuscript and the referee reports from The EMBO Journal. As you know, the referees think that these findings are of interest. However, they have several comments, concerns, and suggestions, indicating that a major revision of the manuscript is necessary to allow publication of the study in EMBO reports.

I would thus like to invite you to revise your manuscript with the understanding that all concerns of the referees must be addressed in the revised manuscript and/or in a detailed point-by-point response. Please also include the textual revisions/expanded discussions of the proposed model as indicated by referee #1.

Acceptance of your manuscript will depend on a positive outcome of a second round of review, involving the same referees. It is EMBO reports policy to allow a single round of revision only and acceptance of the manuscript will therefore depend on the completeness of your responses included in the next, final version of the manuscript.

Revised manuscripts should be submitted within three months of a request for revision. Please contact me to discuss the revision (also by video chat) if you have questions or comments regarding the revision, or should you need additional time.

When submitting your revised manuscript, please also carefully review the instructions that follow below.

PLEASE NOTE THAT upon resubmission revised manuscripts are subjected to an initial quality control prior to exposition to re-review. Upon failure in the initial quality control, the manuscripts are sent back to the authors, which may lead to delays. Frequent reasons for such a failure are the lack of the data availability section (please see below) and the presence of statistics based on n=2 (the authors are then asked to present scatter plots or provide more data points).

When submitting your revised manuscript, we will require:

1) a .docx formatted version of the final manuscript text (including legends for main figures, EV figures and tables), but without the figures included. Figure legends should be compiled at the end of the manuscript text.

2) individual production quality figure files as .eps, .tif, .jpg (one file per figure), of main figures and EV figures. Please upload these as separate, individual files upon re-submission.

The Expanded View format, which will be displayed in the main HTML of the paper in a collapsible format, has replaced the Supplementary information. You can submit up to 6 images as Expanded View. Please follow the nomenclature Figure EV1, Figure EV2 etc. The figure legend for these should be included in the main manuscript document file in a section called Expanded View Figure Legends after the main Figure Legends section. Additional Supplementary material should be supplied as a single pdf file labeled Appendix. The Appendix should have page numbers and needs to include a table of content on the first page (with page numbers) and legends for all content. Please follow the nomenclature Appendix Figure Sx, Appendix Table Sx etc. throughout the text, and also label the figures and tables according to this nomenclature.

For more details, please refer to our guide to authors:
https://link.springer.com/journal/44319/submission-guidelines#cms-Revised-submissions

Please consult our guide for figure preparation:
https://media.springernature.com/original/springer-cms/rest/v1/content/27825798/data/v1

3) a .docx formatted letter INCLUDING the reviewers' reports and your detailed point-by-point responses to their comments. As part of the EMBO Press transparent editorial process, the point-by-point response is part of the Review Process File (RPF), which will be published alongside your paper.

4) a complete author checklist, which you can download from our author guidelines (https://link.springer.com/journal/44319/submission-guidelines#cms-Revised-submissions). Please insert page numbers in the checklist to indicate where the requested information can be found in the manuscript. The completed author checklist will also be part of the RPF.

Please also follow our guidelines for the use of living organisms, and the respective reporting guidelines:
https://link.springer.com/partners/embo-press/editorial-policies#Animal%20and%20human%20studies

5) that primary datasets produced in this study (e.g. RNA-seq, ChIP-seq, structural and array data) are deposited in an appropriate public database. If no primary datasets have been deposited, please also state this in a dedicated section (e.g. 'No primary datasets have been generated and deposited'), see below.

See also: https://link.springer.com/partners/embo-press/editorial-policies#Data%20deposition

Please remember to provide a reviewer password if the datasets are not yet public.

The accession numbers and database should be listed in a formal "Data Availability" section that follows the model below. This is now mandatory (like the COI statement). Please note that the Data Availability Section is restricted to new primary data that are part of this study. This section is mandatory. As indicated above, if no primary datasets have been deposited, please state this in this section

Data availability

The datasets produced in this study are available in the following databases:

- RNA-Seq data: Gene Expression Omnibus GSE46843 (https://www.ncbi.nlm.nih.gov/geo/query/acc.cgi?acc=GSE46843)
- [data type]: [name of the resource] [accession number/identifier/doi] ([URL or identifiers.org/DATABASE:ACCESSION])

*** Note - All links should resolve to a page where the data can be accessed. ***

See also: https://link.springer.com/partners/embo-press/editorial-policies#Data%20availability%20statement

Moreover, I have these editorial requests:

6) We now request the publication of original source data with the aim of making primary data more accessible and transparent to the reader. You will receive a separate email with instructions for providing source data with your revised manuscript, including information how to upload and organize the files.

7) Our journal encourages inclusion of *data citations in the reference list* to directly cite datasets that were re-used and obtained from public databases. Data citations in the article text are distinct from normal bibliographical citations and should directly link to the database records from which the data can be accessed. In the main text, data citations are formatted as follows: "Data ref: Smith et al, 2001" or "Data ref: NCBI Sequence Read Archive PRJNA342805, 2017". In the Reference list, data citations must be labeled with "[DATASET]". A data reference must provide the database name, accession number/identifiers and a resolvable link to the landing page from which the data can be accessed at the end of the reference. Further instructions are available at: https://link.springer.com/journal/44319/submission-guidelines#cms-Reference-guidelines

8) Regarding data quantification and statistics, please make sure that the number "n" for how many independent experiments were performed, their nature (biological versus technical replicates), the bars and error bars (e.g. SEM, SD) and the test used to calculate p-values is indicated in the respective figure legends (also for EV and Appendix figures). Please also check that all the p-values are explained in the legend, and that these fit to those shown in the figure. Please provide statistical testing where applicable. Please avoid the phrase 'independent experiment' but clearly state if these were biological or technical replicates. Please also indicate (e.g. with n.s.) if testing was performed, but the differences are not significant. In case n=2, please show the data as separate datapoints without error bars and statistics. See also: https://link.springer.com/journal/44319/submission-guidelines#cms-Figure-and-data-presentation

If n<5, please show single datapoints for diagrams.

9) Please add scale bars of similar style and thickness to microscopic images, using clearly visible black or white bars (depending on the background). Please place these in the lower right corner of the images themselves. Please do not write on or near the bars in the image but define the size in the respective figure legend.

10) Please also note our reference format: https://link.springer.com/journal/44319/submission-guidelines#cms-Reference-guidelines

11) We updated our journal's competing interests policy in January 2022 and request authors to consider both actual and perceived competing interests. Please review the policy https://www.embopress.org/competing-interests and update your competing interests if necessary. Please name this section 'Disclosure and Competing Interests Statement' and put it after the Acknowledgements section.
See: https://link.springer.com/partners/embo-press/editorial-policies#Competing%20interest%20disclosures

12) We now use CRediT to specify the contributions of each author in the journal submission system. CRediT replaces the author contribution section. Please use the free text box to provide more detailed descriptions and do NOT provide your final manuscript text file with an author contributions section. See also our guide to authors (section 'Author contributions'): https://link.springer.com/journal/44319/submission-guidelines#cms-Revised-submissions

13) All Materials and Methods need to be described in the main text using our 'Structured Methods' format, which is required for all research articles. According to this format, the Methods section should include a Reagents and Tools Table (listing key reagents, experimental models, software, and relevant equipment and including their sources and relevant identifiers), uploaded as separate file, and a Methods section in which we encourage the authors to describe their methods using a step-by-step protocol format with bullet points, to facilitate the adoption of the methodologies across labs. More information on how to adhere to this format as well as downloadable templates (.doc) for the Reagents and Tools Table can be found in our author guidelines (section 'Structured Methods'):

https://link.springer.com/journal/44319/submission-guidelines#cms-Manuscript-organisation-and-formatting

14) Please order the manuscript sections like this, using only these names:
Title page - Abstract (max. 175 words) - Keywords - Introduction - Results - Discussion - Methods - Data availability section - Acknowledgements (please include here all the funding information) - Disclosure and Competing Interests Statement - References - Figure legends - Expanded View Figure legends

Please provide a title with not more than 100 characters (including spaces)

15) Please make sure that all the funding information is also entered into the online submission system and that it is complete and similar to the one in the acknowledgement section of the manuscript text file.

We would also welcome the submission of cover suggestions, or motifs to be used by our Graphics Illustrator in designing a cover.

As part of the EMBO publication's Transparent Editorial Process, EMBO reports publishes online a Review Process File (RPF) to accompany accepted manuscripts. This File will be published in conjunction with your paper and will include the referee reports, your point-by-point response and all pertinent correspondence relating to the manuscript.

You are able to opt out of this by letting the editorial office know (emboreports@embo.org). If you do opt out, the Review Process File link will point to the following statement: "No Review Process File is available with this article, as the authors have chosen not to make the review process public in this case."

I look forward to seeing a revised version of your manuscript when it is ready. Please let me know if you have questions or comments regarding the revision.

Kind regards,

Achim

Achim Breiling
Senior Editor
EMBO Reports

Referee #1:

The manuscript describes the structure of WNV with a an ultra potent Fab fragment bound to it. The model the authors describes poses more questions than it answers. The authors claim the
structure reveals the mechanism of neutralization - is this really true ? I think the claims are overblown. What the model does do is define the quaternary epitope of the Fab and supports a model in which dimer locking blocks the fusion-triggering rearrangements, consistent with prior functional data. The authors infer the mechanism but it is not experimentally pinned down -

I found the paper difficult to read and much of the language is imprecise, sometime verging

The text refers to "involve residues across the dimer two-fold axis" - WNV-86 uses an inter-protomer, off-axis epitope that depends on the two-fold geometry, but it doesn't sit on the axis itself.

Another example is "the E dimer near the two-fold axis, which is also the site for prM binding." The 2-fold axis is defined symmetry relationship - not a binding site and prM doesn't bind to residues on E that are situated on or close to the 2-fold axis.

Again - "Three loops from prM, are important in occluding WNV-86 binding:
residues on HK-loop could clash with those on LCDR3 loop and HC Fv domain β strands 4 and 5; prM β 4/5 and β 5/6 loops

would sterically hinder HCDR1 and HCDR3 (Fig. 5A)."
The model suggests the Ab would not be able to bind if prM is present due to steric clashing.

What does "HCDR3 loop is approx. 5.2 Å wide, whereas the E dimer width is about 10.7 Å " mean ?? How is this width defined ?? What does the width of the E dimer mean ?

The positioning of the HCDR3 in the dimer groove is unlikely to be a fortuitous occurrence - I agree it will be down to the energetics of binding of the FAB CDRs to that region.

Finally, the structure poses some questions that aren't addressed satisfactorily. Why does the Fab not bind to the 2-fold related epitope so that you get a mixture of partially occupied sites ? Why don't you get the Fab binding to the an epitope on the icosahedral E dimer ??
The data processing in the methods section is not described clearly enough. Did the authors try any focused classification to pull out complexes where Fabs are binding to these alternative sites ??
Did the authors try incubating the complex at 30 deg, given that we know the E capsid is dynamic - it may allow flexing to allow better binding with higher occupancy.

Referee #2:

In "A distinct E dimer epitope underlies selective recognition by a protective human West Nile virus antibody", Khare, Crowe, Kuhn, and colleagues determine the cryo-EM structure of the antigen-binding fragment (Fab) of WNV-86, an ultrapotent protective human monoclonal antibody targeting the West Nile virus (WNV) E protein, which preferentially recognizes mature virions - in complex with WNV particles (KUNV virus, strain MRM61C). The authors encounter low occupancy, in part because of steric incompatibility of WNV-86 binding to all potential binding sites on the WNV particle and focus their description on Fab recognition of the general E dimer.

The authors state that WNV-86 bound in a single preferred unidirectional manner - which they model in Fig. 1B. It's unclear to me why one orientation is preferred versus the other; it would be helpful for the authors to discuss/clarify why the single orientation was preferred - and if this were true of all particles or if some particles were occupied with WNV-86 bound in an alternative manner.

Despite the low resolution, the authors define the WNV-86 epitope and HCDR loop in detail and compare the WNV-86 epitope with epitopes of other known intra-dimer antibodies, such as ZIKV DH1017 or those from DENV. The authors also detail the overlap of WNV-86 binding with prM binding. The authors also define residues involved in escape from WNV-86, including glycosylation. Lastly, the authors pull all of this information together to define a new class of EDE antibodies - class 3 - with six particular characteristics.

Overall, the authors do a nice job describing the structure of WNV-86. I didn't see any discussion on the binding of IgG; the authors should comment on whether simultaneous binding of the two Fab arms of bivalent IgG can be accommodated - and integrate this observation into what has been observed functionally for Fab versus IgG neutralization by WNV-86.

Minor revisions the authors should address:
1. Abstract last sentence. Grammar. "The structure reveals... and clarifies..." In the reveal part, it should be "the mechanism of... and the role of...
2. The authors should be careful to restrict their use of present tense in describing their results to only when such statements are generally true. For example, on line 127, the occupancy result is stated in present tense. this should be in past tense (e.g., The occupancy of the WNV-86 Fab was low"; while the result is correct for the complex determined, the occupancy of antibodies in cryo-EM complexes can vary depending on experiment conditions of complex formation and freezing - so it may not always be low. Similarly on line 129 - "The density at the general dimer position showed... (not shows), and for the rest of the results.
3. (line 133) The authors state that "Localized reconstruction of the ASU failed to improve resolution. The authors should clarify if they assessed asymmetric units by peak picking/classification to only average those asymmetric units with similarly bound Fab, as such peak peaking/classification has assisted in increasing density in other antibody-virus complexes.

Referee #1:

The manuscript describes the structure of WNV with a an ultra potent Fab fragment bound to it. The model the authors describes poses more questions than it answers. The authors claim the structure reveals the mechanism of neutralization - is this really true ? I think the claims are overblown. What the model does do is define the quaternary epitope of the Fab and supports a model in which dimer locking blocks the fusion-triggering rearrangements, consistent with prior functional data. The authors infer the mechanism but it is not experimentally pinned down

We thank the reviewer for the careful feedback. Although our analysis goes much beyond description of an epitope, notably shedding light on the structural basis of the mutational data acquired by Goo *et al*., 2019 and bringing specific insights, it does not fully describe the neutralization mechanism. We addressed the reviewer concern accordingly and revised mention of “a neutralization mechanism” in the Abstract, Introduction and Discussion sections to accurately reflect the data and its interpretation as noted above.

The end of the abstract now reads as “The structure reveals the quaternary epitope of the neutralizing Fab and supports a model in which engaging both protomers of the dimer likely interferes with the fusion-triggering rearrangements. The studies identify critical residues for binding, neutralization and immune escape, and clarify the promise of this molecule for future immunotherapeutic interventions”.

I found the paper difficult to read and much of the language is imprecise, sometime verging The text refers to "involve residues across the dimer two-fold axis" - WNV-86 uses an inter-

protomer, off-axis epitope that depends on the two-fold geometry, but it doesn't sit on the axis itself.

Another example is "the E dimer near the two-fold axis, which is also the site for prM binding." The 2-fold axis is defined symmetry relationship - not a binding site and prM doesn't bind to residues on E that are situated on or close to the 2-fold axis.

Thank you for pointing this out and the specific examples. We have revised all statements involving the mention of the two-fold axis in the manner pointed out by the Reviewer and have rewritten other sections.

Again - "Three loops from prM, are important in occluding WNV-86 binding: residues on HK-loop could clash with those on LCDR3 loop and HC Fv domain β strands 4 and 5; prM β 4/5 and β 5/6 loops would sterically hinder HCDR1 and HCDR3 (Fig. 5A)." The model suggests the Ab would not be able to bind if prM is present due to steric clashing.

Thank you for the comment. We have revised the sentence for clarity and precision, and we have retained the specific description of the three loops involved in steric clashing.

What does "HCDR3 loop is approx. 5.2 Å wide, whereas the E dimer width is about 10.7 Å " mean ?? How is this width defined ?? What does the width of the E dimer mean?

Thank you for pointing out this statement. The distance between two Cα atoms, from Gly105 and Thr108 in the loop, was measured to be approx. 5.2 Å; this was the widest distance between Cα atoms from two juxtaposing residues in the loop. In the dimer, the distance between the closest

Cα atoms from residues in the two chains of the dimer (residues Lys209 on chain C and Ala254 on chain E) was measured to be 10.7 Å. We have revised the sentence in the text and the figure legend to describe this point clearly.

The positioning of the HCDR3 in the dimer groove is unlikely to be a fortuitous occurrence - I agree it will be down to the energetics of binding of the FAB CDRs to that region.

Finally the structure poses some questions that aren't addressed satisfactorily. Why does the Fab not bind to the 2-fold related epitope so that you get a mixture of partially occupied sites? Why don't you get the Fab binding to an epitope on the icosahedral E dimer ??
The data processing in the methods section is not described clearly enough. Did the authors try any focused classification to pull out complexes where Fabs are binding to these alternative sites??

Thank you for the thoughtful questions. We agree with the Reviewer's two points above: 1) ". . . it will be down to the energetics of the binding of the Fab CDRs to that region . . ." and 2) "the structure poses some questions that aren't addressed satisfactorily". Indeed, the findings of our investigations provide insights that raise several questions regarding the binding of the Fab at the three sites in the asymmetric subunit. We have now added in the Discussion a description of the structural basis for the preferential binding at site 1. For this, we first defined the epitope to comprise of two parts (residing on separate envelope glycoprotein chains of a dimer, epitope segment I and II). We then show the presence of a shift in the β strands at site 2, epitope segment II, which leads to clashes between atoms of the envelope glycoprotein and the Fv HC residues. A few clashes involve Cα or Cβ atoms which would abrogate binding at site 2. The differences in

the β strands are also present in the native WNV chains, possibly due to the non-equivalence of the chains in the ASU, suggesting that site 1 is a more favorable site for binding. A mixture of the binding at sites 1 and 2 or partially occupied sites is not observed because: 1) the two sites are non-equivalent and site 2 displays clashes with a modeled Fab at this site, and 2) by definition of EDE3 antibodies, once site 1 is occupied, steric hindrance abrogates binding at site 2 completely. We also show that the close contact distances at the icosahedral site are intermediate between site 1 and site 2 (a table is included) or similar to that at site 1, therefore site 3 could potentially support binding. The distances between interfacing residues of the glycoprotein and the Fv HC domain correlate with the variation in densities for the Fab observed at the three sites.

Our reconstruction showed that the density at the three sites varies: site 1 shows clear density for the Fv domain of the Fab heavy chain and the Fv domains of both the light and heavy chains of the Fab were modeled. According to our 3D classification results this site is occupied in around 30% of all asymmetric units. Site 2 is the two-fold related site on the general E dimer where density is not observed at all despite multiple classification attempts; binding at this site would sterically clash with a Fab bound at site 1. Site 3 has the E dimer with its two-fold coinciding with the icosahedral two-fold axis. There is some density at site 3; however, this is not sufficient for confidently fitting in the Fv domains of the heavy or light chains of WNV-86 Fab albeit enough to confirm that the Fab does bind here as well. 3D classification showed Fab occupancies of ~10% for both possible orientations. We have added these clarifications in the Methods section.

We have revised the draft and added text in the Discussion sections per the Reviewer's suggestions.

Did the authors try incubating the complex at 30 deg, given that we know the E capsid is dynamic - it may allow flexing to allow better binding with higher occupancy.

Thank you for this comment. The present research did not aim to clarify occupancy of the Fab binding at different temperatures. The complex formation for WNV-86 Fab was carried out at 37ºC, which is where one would expect to observe higher dynamics for differential epitope engagement compared with a lower temperature.

Referee #2:

In "A distinct E dimer epitope underlies selective recognition by a protective human West Nile virus antibody", Khare, Crowe, Kuhn, and colleagues determine the cryo-EM structure of the antigen-binding fragment (Fab) of WNV-86, an ultrapotent protective human monoclonal antibody targeting the West Nile virus (WNV) E protein, which preferentially recognizes mature virions - in complex with WNV particles (KUNV virus, strain MRM61C). The authors encounter low occupancy, in part because of steric incompatibility of WNV-86 binding to all potential binding sites on the WNV particle and focus their description on Fab recognition of the general E dimer.

The authors state that WNV-86 bound in a single preferred unidirectional manner - which they model in Fig. 1B. It's unclear to me why one orientation is preferred versus the other; it would be helpful for the authors to discuss/clarify why the single orientation was preferred - and if this were true of all particles or if some particles were occupied with WNV-86 bound in an alternative manner.

Thank you for pointing this out. We have revised the text to state that WNV-86 Fab displays preferential binding at site 1. We have included in the Discussion the structural basis for this

preferential binding. Briefly, structural differences at site 2 are unfavorable for binding: inherent differences between the non-equivalent glycoprotein chains manifests in a shift in some β strands at site 2. This shift leads to clashes of specific residues with those of the HC Fv domain. Additionally, binding at site 1 would abrogate binding at site 2 due to steric hindrance. Our classification shows a 30% occupancy at one of the general sites and around 10% occupancy each for the two different binding sites at the icosahedral axes.

Despite the low resolution, the authors define the WNV-86 epitope and HCDR loop in detail and compare the WNV-86 epitope with epitopes of other known intra-dimer antibodies, such as ZIKV DH1017 or those from DENV. The authors also detail the overlap of WNV-86 binding with prM binding. The authors also define residues involved in escape from WNV-86, including glycosylation. Lastly, the authors pull all of this information together to define a new class of EDE antibodies - class 3 - with six particular characteristics.

Overall, the authors do a nice job describing the structure of WNV-86. I didn't see any discussion on the binding of IgG; the authors should comment on whether simultaneous binding of the two Fab arms of bivalent IgG can be accommodated - and integrate this observation into what has been observed functionally for Fab versus IgG neutralization by WNV-86.

Thank you for the thoughtful comment. A comparison of the Fab binding with IgG was not pursued; however, we have now included a discussion on the distances between sites 1-3 within the raft and adjacent sites that could potentially accommodate IgG binding. Based on these distances, it is possible that an IgG antibody can bind to any two adjacent sites 1 or 3. However, given the differential binding of the Fab at sites 1-3, non-identical dynamics of IgG Fv domains

and the peculiar placement of the HCDR3 loop at site 1 observed for WNV-86 Fab, a theoretical discussion on bivalent binding is highly speculative. Energetics and dynamics at the protein interfaces could contribute to differences in binding. Hence, experimental investigations of the structure of WNV with IgG are needed to correlate observed binding sites with IgG with the currently available functional data of Fab vs IgG neutralization.

As the Reviewer appreciated, given the limited resolution of the reconstruction, extracting meaningful information that bridges structural changes with functional consequences would be additionally challenging. To clarify the Reviewer's questions, we have now included a detailed discussion of the limitations of the research in the Discussion section.

Minor revisions the authors should address:

1. Abstract last sentence. Grammar. "The structure reveals... and clarifies..." In the reveal part, it should be "the mechanism of... and the role of...

Thank you for pointing this out. Along the lines of this suggestion and a comment from Reviewer #1, the last line in the Abstract is revised and corrected.

2. The authors should be careful to restrict their use of present tense in describing their results to only when such statements are generally true. For example, on line 127, the occupancy result is stated in present tense. this should be in past tense (e.g., The occupancy of the WNV-86 Fab was low"; while the result is correct for the complex determined, the occupancy of antibodies in cryo-EM complexes can vary depending on experiment conditions of complex formation and freezing - so it may not always be low. Similarly on

line 129 - "The density at the general dimer position showed... (not shows), and for the rest of the results.

We thank the Reviewer for this comment. All instances of the issue have been revised in the Results section.

3. (line 133) The authors state that "Localized reconstruction of the ASU failed to improve resolution. The authors should clarify if they assessed asymmetric units by peak picking/classification to only average those asymmetric units with similarly bound Fab, as such peak peaking/classification has assisted in increasing density in other antibody-virus complexes.

Thank you for this helpful comment. The final localized reconstruction included 52.9% of all asymmetric subunits; focused classification helped to get an approximation of the occupancy at each site in the asymmetric subunit, but did not improve the resolution or the quality of the map, as it was already close to the Nyquist limit of the dataset and likely because of the reduced number of particles that were included in the final localized reconstructions. A notable limitation of the present research is the limited number of particles and pixel size at which data were collected, the best detector available at the time. We have now revised the draft to clarify these points in the Methods and Discussion section.

Dear Dr. Kuhn,

Thank you for the submission of your revised manuscript to our editorial offices. I have now received the reports from the two referees that were asked to re-evaluate the study, you will find below. As you will see, the referees now support the publication of the study in EMBO reports. Both referees have remaining minor concerns and suggestions to improve the manuscript I ask you to address in a final revised version. Please also provide a final p-b-p-response to these points and the editorial requests below.

Editorial requests:

- Please order the manuscript sections like this, using only these names:
Title page - Abstract - Keywords - Introduction - Results - Discussion - Methods - Data availability section - Acknowledgements (please include here all the funding information) - Disclosure and Competing Interests Statement - References - Figure legends - Expanded View Figure legends

Please move the affiliations to the title page and indicate the corr. Author, including an email address on the title page.

It seems the 'Ethics Declaration' presently provided is the Disclosure and Competing Interests Statement. Please change the name and put it at the right place (see above).

- There are several empty pages in the manuscript text file. Please remove these and also provide the final text file as clean manuscript (without the notes on changes).

- We now use CRediT to specify the contributions of each author in the journal submission system. CRediT replaces the author contribution section. Please use the free text box to provide more detailed descriptions and do NOT provide your final manuscript text file with an author contributions section. See also our guide to authors (section 'Author contributions'):
https://link.springer.com/journal/44319/submission-guidelines#cms-Revised-submissions

- Please add direct links for the datasets indicated in the Data Availability section (EMDB and PDB IDs: EMD-48850 and 9N3B) and make sure these are public latest upon online publication of the study.

- Please make sure that each figure panel is called out separately and that the panels are called out sequentially. Presently, a Fig. 7 is called out, but no such figure has been uploaded. Please check.

- A Table S1is called out. I guess this is 'Appendix Table S1'. Please add the word Appendix.

- Please add a title page to the Appendix with "Appendix to [Title of the ms]" and a table of contents with each item listed with its page number. The panel labels for each figure must not have "Appendix Sx" in the name, just the letters. The correct nomenclature for the names of the Appendix items throughout the file should be "Appendix Figure S1x' and 'Appendix Table Sx'. Please correct this.

All Materials and Methods need to be described in the main text using our 'Structured Methods' format, which is required for all research articles. According to this format, the Methods section should include a Reagents and Tools Table (listing key reagents, experimental models, software, and relevant equipment and including their sources and relevant identifiers), uploaded as separate file, and a Methods section in which we encourage the authors to describe their methods using a step-by-step protocol format with

bullet points, to facilitate the adoption of the methodologies across labs. More information on how to adhere to this format as well as downloadable templates (.doc) for the Reagents and Tools Table can be found in our author guidelines (section 'Structured Methods'):

https://link.springer.com/journal/44319/submission-guidelines#cms-Manuscript-organisation-and-formatting

- Thanks for providing the source data. Please upload the source data as one folder per main figure, grouping together the separate files for the panels (clearly labelled), and ZIPed together, and one folder for the EV figures, grouping together the separate files for the EV figures (clearly labelled), and ZIPed together. Please also upload a fully compiled source data checklist with your final submission.

In addition, I would need from you uploaded separately:
- a short, two-sentence summary of the manuscript (not more than 35 words).
- two to four short (!) bullet points highlighting the key findings of your study (two lines each).
- a schematic summary figure as separate file that provides a sketch of the major findings (not a data image) in jpeg or tiff format (with the exact width of 550 pixels and the exact height of 300 pixels) that can be used as a visual synopsis on our website.

I look forward to seeing the further revised version of your manuscript when it is ready. Please let me know if you have questions regarding the revision.

Best,

Achim Breiling
Senior Editor
EMBO Reports

Referee #1:

The manuscript by Khare et al. provides a nice description of the cryo-EM structure of WNV-86, an ultrapotent antibody that binds to a new class of E dimers.

In general, the authors have responded robustly to my and the other reviewer's comments.

My only remaining comment is that the discussion does a nice job touching on the various ways the structure provides insight, but I didn't see an explanation for the ultra-potency of WNV-86; it would be helpful for the authors to provide.

Referee #2:

The revised manuscript is substantially improved and the authors have addressed the main concern that the original submission overclaimed a demonstrated neutralization mechanism. The revised abstract and parts of the discussion now more appropriately describe the structure as defining a quaternary epitope

and supporting a plausible model for inhibition of fusion-triggering rearrangements. The added occupancy/focused-classification information and discussion of site preference are also helpful.

However, some statements in the Discussion still present structural interpretations as if they were experimentally demonstrated mechanisms. I recommend further softening phrases such as "confers selective recognition," "is essential for neutralization," and related statements about tetramer stability and neutralization, unless directly supported by experiments in the present study. The proposed EDE3 classification is reasonable, but should remain explicitly framed as a proposed subclass based on the present structure and comparison with related antibodies.

Referee #1:

Referee #1:

The manuscript by Khare et al. provides a nice description of the cryo-EM structure of WNV-86, an ultrapotent antibody that binds to a new class of E dimers.

In general, the authors have responded robustly to my and the other reviewer's comments. My only remaining comment is that the discussion does a nice job touching on the various ways the structure provides insight, but I didn't see an explanation for the ultra-potency of WNV-86; it would be helpful for the authors to provide.

We thank the reviewer for this comment. A structure can provide unique insights into molecular differences and fine details that may significantly affect potency. However, definitive determination of the structural basis of ultrapotent neutralization by an antibody requires complementary experimental evidence and comparative analyses. Further, the resolution of the present complex of WNV virus and the WNV-86 Fab restricts a meaningful understanding of the residue-level structural details of interactions that may contribute to the ultrapotent neutralization by WNV-86. Nonetheless, we agree with the reviewer that this is an important point that merits inclusion therefore we have now added a few sentences at the end of Discussion to include some of associated points.

Referee #2:

The revised manuscript is substantially improved and the authors have addressed the main

concern that the original submission overclaimed a demonstrated neutralization mechanism. The revised abstract and parts of the discussion now more appropriately describe the structure as defining a quaternary epitope and supporting a plausible model for inhibition of fusion-triggering rearrangements. The added occupancy/focused-classification information and discussion of site preference are also helpful.

However, some statements in the Discussion still present structural interpretations as if they were experimentally demonstrated mechanisms. I recommend further softening phrases such as "confers selective recognition," "is essential for neutralization," and related statements about tetramer stability and neutralization, unless directly supported by experiments in the present study. The proposed EDE3 classification is reasonable but should remain explicitly framed as a proposed subclass based on the present structure and comparison with related antibodies.

We thank the reviewer for the important comment and appreciate the attention to detail. We have reviewed and/or revised phrases in Discussion that are not directly supported by experimental evidence, especially for any references to a neutralization mechanism. Specifically -

- We revised the previous statement mentioning “is essential for neutralization” and provided more context. We revised other phrases mentioned above to better reflect the alignment with experimental data.
- We revised statements mentioning the role of tetramer stability to better reflect the point in context of the available structural data.
- We revised several words throughout the Discussion, to add context or considering whether the current structural data supports the statement.

- We retained the proposed EDE3 classification and EDE-like antibodies as a subclass, as supported by the present structural investigation and stated limitations therein.

2nd Revision - Editorial Decision 17th Jun 2026

Dr. Richard J. Kuhn
Purdue University
Department of Biological Sciences
HOCK 225
West Lafayette 47907-2054
United States

Dear Dr. Kuhn,

Thank you for the submission of your final revised manuscript to our editorial offices. I now went through this and your final p-b-p-response and consider the remaining points of the referees and the editorial requests as adequately addressed.

I am thus very pleased to accept your manuscript for publication in the next available issue of EMBO reports. Thank you for your contribution to our journal.

Your manuscript will be processed for publication by EMBO Press. It will be copy edited and you will receive page proofs prior to publication. Please note that you will be contacted by Springer Nature Author Services to complete licensing and payment information.

You may qualify for financial assistance for your publication charges - either via a Springer Nature fully open access agreement or an EMBO initiative. Check your eligibility: https://link.springer.com/journal/44319/how-to-publish-with-us

Should you be planning a Press Release on your article, please notify the production team via this form in order to coordinate publication and release dates: https://forms.cloud.microsoft/e/eH4A8JVP6g

If you have any questions, please do not hesitate to contact the Editorial Office. Thank you for your contribution to EMBO Reports.

Yours sincerely,

Achim Breiling
Senior Editor
EMBO Reports

>>> Please note that it is EMBO Reports policy for the transcript of the editorial process (containing referee reports and your response letter) to be published as an online supplement to each paper. If you do NOT want this, you will need to inform the Editorial Office via email immediately. More information is available here: https://link.springer.com/partners/embo-press/editorial-policies#Peer%20review

EMBO Press Author Checklist

Corresponding Author Name: Richard J. Kuhn	
Journal Submitted to: EMBO Reports	
Manuscript Number: EMBOR-2025-63450V2	

USEFUL LINKS FOR COMPLETING THIS FORM
The EMBO Journal - Author Guidelines
EMBO Reports - Author Guidelines
Molecular Systems Biology - Author Guidelines
EMBO Molecular Medicine - Author Guidelines

Reporting Checklist for Life Science Articles (updated January
This checklist is adapted from Materials Design Analysis Reporting (MDAR) Checklist for Authors. MDAR establishes a minimum set of requirements in transparent reporting in the life sciences (see Statement of Task: 10.31222/osf.io/9sm4x). Please follow the journal's guidelines in preparing your
Please note that a copy of this checklist will be published alongside your article.

Abridged guidelines for figures
1. Data
The data shown in figures should satisfy the following conditions:
- the data were obtained and processed according to the field's best practice and are presented to reflect the results of the experiments in an accurate and unbiased manner.
- ideally, figure panels should include only measurements that are directly comparable to each other and obtained with the same assay.
- plots include clearly labeled error bars for independent experiments and sample sizes. Unless justified, error bars should not be shown for technical
- if n<5, the individual data points from each experiment should be plotted. Any statistical test employed should be justified.
- Source Data should be included to report the data underlying figures according to the guidelines set out in the authorship guidelines on Data

2. Captions
Each figure caption should contain the following information, for each panel where they are relevant:
- a specification of the experimental system investigated (eg cell line, species name).
- the assay(s) and method(s) used to carry out the reported observations and measurements.
- an explicit mention of the biological and chemical entity(ies) that are being measured.
- an explicit mention of the biological and chemical entity(ies) that are altered/varied/perturbed in a controlled manner.
- the exact sample size (n) for each experimental group/condition, given as a number, not a range;
- a description of the sample collection allowing the reader to understand whether the samples represent technical or biological replicates (including how many animals, litters, cultures, etc.).
- a statement of how many times the experiment shown was independently replicated in the laboratory.
- definitions of statistical methods and measures:
  - common tests, such as t-test (please specify whether paired vs. unpaired), simple χ2 tests, Wilcoxon and Mann-Whitney tests, can be unambiguously identified by name only, but more complex techniques should be described in the methods section;
  - are tests one-sided or two-sided?
  - are there adjustments for multiple comparisons?
  - exact statistical test results, e.g., P values = x but not P values < x;
  - definition of 'center values' as median or average;
  - definition of error bars as s.d. or s.e.m.

Please complete ALL of the questions below.
Select "Not Applicable" only when the requested information is not relevant for your study.

Materials

Newly Created Materials	Information included in the manuscript?	In which section is the information available? (Reagents and Tools Table, Materials and Methods, Figures, Data Availability Section)
New materials and reagents need to be available; do any restrictions apply?	Not Applicable	

Antibodies	Information included in the manuscript?	In which section is the information available? (Reagents and Tools Table, Materials and Methods, Figures, Data Availability Section)
For **antibodies** provide the following information:<br>- Commercial antibodies: RRID (if possible) or supplier name, catalogue number and or/clone number<br>- Non-commercial: RRID or citation	Not Applicable	

DNA and RNA sequences	Information included in the manuscript?	In which section is the information available? (Reagents and Tools Table, Materials and Methods, Figures, Data Availability Section)
**Short novel DNA or RNA including primers, probes**: provide the sequences.	Not Applicable	

Cell materials	Information included in the manuscript?	In which section is the information available? (Reagents and Tools Table, Materials and Methods, Figures, Data Availability Section)
**Cell lines:** Provide species information, strain. Provide accession number in repository **OR** supplier name, catalog number, clone number, and/**OR** RRID.	Not Applicable	
**Primary cultures:** Provide species, strain, sex of origin, genetic modification status.	Not Applicable	
Report if the cell lines were recently **authenticated** (e.g., by STR profiling) and tested for mycoplasma contamination.	Not Applicable	

Experimental animals	Information included in the manuscript?	In which section is the information available? (Reagents and Tools Table, Materials and Methods, Figures, Data Availability Section)
**Laboratory animals or Model organisms:** Provide species, strain, sex, age, genetic modification status. Provide accession number in repository **OR** supplier name, catalog number, clone number, **OR** RRID.	Not Applicable	
**Animal observed in or captured from the field:** Provide species, sex, and age where possible**.**	Not Applicable	
Please detail **housing and husbandry conditions**.	Not Applicable	

Plants and microbes	Information included in the manuscript?	In which section is the information available? (Reagents and Tools Table, Materials and Methods, Figures, Data Availability Section)
**Plants:** provide species and strain, ecotype and cultivar where relevant, unique accession number if available, and source (including location for collected wild specimens).	Not Applicable	
**Microbes:** provide species and strain, unique accession number if available, and source.	Not Applicable	

Human research participants	Information included in the manuscript?	In which section is the information available? (Reagents and Tools Table, Materials and Methods, Figures, Data Availability Section)
If collected and within the bounds of privacy constraints report on age, sex and gender or ethnicity for all study participants.	Not Applicable	

Core facilities	Information included in the manuscript?	In which section is the information available? (Reagents and Tools Table, Materials and Methods, Figures, Data Availability Section)
If your work benefited from core facilities, was their service mentioned in the acknowledgments section?	Yes	Data availability

Design

Study protocol	Information included in the manuscript?	In which section is the information available? (Reagents and Tools Table, Materials and Methods, Figures, Data Availability Section)
If study protocol has been **pre-registered, provide DOI in the manuscript**. For clinical trials, provide the trial registration number **OR** cite DOI.	Not Applicable	
Report the **clinical trial registration number** (at ClinicalTrials.gov or equivalent), where applicable.	Not Applicable	

Laboratory protocol	Information included in the manuscript?	In which section is the information available? (Reagents and Tools Table, Materials and Methods, Figures, Data Availability Section)
Provide DOI OR other citation details if **external detailed step-by-step protocols** are available.	Not Applicable	

Experimental study design and statistics	Information included in the manuscript?	In which section is the information available? (Reagents and Tools Table, Materials and Methods, Figures, Data Availability Section)
Include a statement about **sample size** estimate even if no statistical methods were used.	Not Applicable	
Were any steps taken to minimize the effects of subjective bias when allocating animals/samples to treatment (e.g. **randomization procedure**)? If yes, have they been described?	Not Applicable	
Include a statement about **blinding** even if no blinding was done.	Not Applicable	
Describe **inclusion/exclusion criteria** if samples or animals were excluded from the analysis. Were the criteria pre-established?<br><br>If sample or data points were omitted from analysis, report if this was due to attrition or intentional exclusion and provide justification.	Not Applicable	
For every figure, are **statistical tests** justified as appropriate? Do the data meet the assumptions of the tests (e.g., normal distribution)? Describe any methods used to assess it. Is there an estimate of variation within each group of data? Is the variance similar between the groups that are being statistically compared?	Not Applicable	

Sample definition and in-laboratory replication	Information included in the manuscript?	In which section is the information available? (Reagents and Tools Table, Materials and Methods, Figures, Data Availability Section)
In the figure legends: state number of times the experiment was **replicated** in laboratory.	Not Applicable	
In the figure legends: define whether data describe **technical or biological replicates**.	Not Applicable	

Ethics

Ethics	Information included in the manuscript?	In which section is the information available? (Reagents and Tools Table, Materials and Methods, Figures, Data Availability Section)
Studies involving **human participants**: State details of **authority granting ethics approval** (IRB or equivalent committee(s), provide reference number for approval.	Not Applicable	
Studies involving **human participants**: Include a statement confirming that **informed consent** was obtained from all subjects and that the experiments conformed to the principles set out in the WMA Declaration of Helsinki and the Department of Health and Human Services Belmont Report.	Not Applicable	
Studies involving **human participants:** For publication of **patient photos**, include a statement confirming that consent to publish was obtained.	Not Applicable	
Studies involving experimental **animals**: State details of **authority granting ethics approval** (IRB or equivalent committee(s), provide reference number for approval. Include a statement of compliance with ethical regulations.	Not Applicable	
Studies involving **specimen and field samples:** State if relevant **permits** obtained, provide details of authority approving study; if none were required, explain why.	Not Applicable	

Dual Use Research of Concern (DURC)	Information included in the manuscript?	In which section is the information available? (Reagents and Tools Table, Materials and Methods, Figures, Data Availability Section)
Could your study fall under dual use research restrictions? Please check biosecurity documents and list of **select agents and toxins** (CDC): https://www.selectagents.gov/sat/list.htm	Not Applicable	
If you used a select agent, is the security level of the lab appropriate and reported in the manuscript?	Not Applicable	
If a study is subject to dual use research of concern regulations, is the name of the **authority granting approval and reference number** for the regulatory approval provided in the manuscript?	Not Applicable	

Reporting

The MDAR framework recommends adoption of discipline-specific guidelines, established and endorsed through community initiatives. Journals have their own policy about requiring specific guidelines and recommendations to complement MDAR.

Adherence to community standards	Information included in the manuscript?	In which section is the information available? (Reagents and Tools Table, Materials and Methods, Figures, Data Availability Section)
State if relevant guidelines or checklists (e.g., **ICMJE, MIBBI, ARRIVE, PRISMA**) have been followed or provided.	Not Applicable	
For **tumor marker prognostic studies,** we recommend that you follow the **REMARK** reporting guidelines (see link list at top right). See author guidelines, under 'Reporting Guidelines'. Please confirm you have followed these guidelines.	Not Applicable	
For **phase II and III randomized controlled trials**, please refer to the **CONSORT** flow diagram (see link list at top right) and submit the CONSORT checklist (see link list at top right) with your submission. See author guidelines, under 'Reporting Guidelines'. Please confirm you have submitted this list.	Not Applicable	

Data Availability

Data availability	Information included in the manuscript?	In which section is the information available? (Reagents and Tools Table, Materials and Methods, Figures, Data Availability Section)
Have **primary datasets** been deposited according to the journal's guidelines (see 'Data Deposition' section) and the respective accession numbers provided in the Data Availability Section?	Not Applicable	
Were **human clinical and genomic datasets** deposited in a public access-controlled repository in accordance to ethical obligations to the patients and to the applicable consent agreement?	Not Applicable	
Are **computational models** that are central and integral to a study available without restrictions in a machine-readable form? Were the relevant accession numbers or links provided?	Not Applicable	
If publicly available data were reused, provide the respective **data citations in the reference list**.	Not Applicable	
